# Brain lesions disrupting addiction map to a common human brain circuit

**DOI:** 10.1038/s41591-022-01834-y

**Published:** 2022-06-13

**Authors:** Juho Joutsa, Khaled Moussawi, Shan H. Siddiqi, Amir Abdolahi, William Drew, Alexander L. Cohen, Thomas J. Ross, Harshawardhan U. Deshpande, Henry Z. Wang, Joel Bruss, Elliot A. Stein, Nora D. Volkow, Jordan H. Grafman, Edwin van Wijngaarden, Aaron D. Boes, Michael D. Fox

**Affiliations:** 1 Turku Brain and Mind Center, Clinical Neurosciences, University of Turku, Turku, Finland; 2 Neurocenter and Turku PET Center, Turku University Hospital, Turku, Finland; 3 Berenson-Allen Center for Noninvasive Brain Stimulation, Beth Israel Deaconess Medical Center, Boston, MA USA; 4 National Institute on Drug Abuse-Intramural Research Program, Baltimore, MD USA; 5 Department of Psychiatry, University of Pittsburgh, Pittsburgh, PA USA; 6 Center for Brain Circuit Therapeutics, Departments of Neurology Psychiatry and Radiology, Brigham and Women’s Hospital, Harvard Medical School, Boston, MA USA; 7 Clinical Affairs, Philips Healthcare, Cambridge, MA USA; 8 Department of Neurology, Boston Children’s Hospital, Boston, MA USA; 9 Computational Radiology Laboratory, Department of Radiology, Boston Children’s Hospital, Harvard Medical School, Boston, MA USA; 10 Department of Imaging Sciences, University of Rochester Medical Center, Rochester, NY USA; 11 Departments of Pediatrics, Neurology & Psychiatry, University of Iowa Hospitals and Clinics, Iowa City, IA USA; 12 Shirley Ryan AbilityLab, Chicago, IL USA; 13 Department of Physical Medicine and Rehabilitation, Neurology, Cognitive Neurology and Alzheimer’s Center, Northwestern University, Chicago, IL USA; 14 Department of Psychiatry, Feinberg School of Medicine and Department of Psychology, Weinberg College of Arts and Sciences, Northwestern University, Chicago, IL USA; 15 Department of Public Health Sciences, University of Rochester Medical Center, Rochester, NY USA; 16 Intramural Research Program, National Institute of Alcohol Abuse and Alcoholism, Bethesda, MD USA

**Keywords:** Brain injuries, Addiction

## Abstract

Drug addiction is a public health crisis for which new treatments are urgently needed. In rare cases, regional brain damage can lead to addiction remission. These cases may be used to identify therapeutic targets for neuromodulation. We analyzed two cohorts of patients addicted to smoking at the time of focal brain damage (cohort 1 *n* = 67; cohort 2 *n* = 62). Lesion locations were mapped to a brain atlas and the brain network functionally connected to each lesion location was computed using human connectome data (*n* = 1,000). Associations with addiction remission were identified. Generalizability was assessed using an independent cohort of patients with focal brain damage and alcohol addiction risk scores (*n* = 186). Specificity was assessed through comparison to 37 other neuropsychological variables. Lesions disrupting smoking addiction occurred in many different brain locations but were characterized by a specific pattern of brain connectivity. This pattern involved positive connectivity to the dorsal cingulate, lateral prefrontal cortex, and insula and negative connectivity to the medial prefrontal and temporal cortex. This circuit was reproducible across independent lesion cohorts, associated with reduced alcohol addiction risk, and specific to addiction metrics. Hubs that best matched the connectivity profile for addiction remission were the paracingulate gyrus, left frontal operculum, and medial fronto-polar cortex. We conclude that brain lesions disrupting addiction map to a specific human brain circuit and that hubs in this circuit provide testable targets for therapeutic neuromodulation.

## Abstract (web summary)

Lesions resulting in addiction remission occur in multiple different brain locations but map to a specific brain circuit.

## Main

Substance use disorders (SUDs) affect 8–10% of the adult population, are a leading cause of death in the young and are considered a public health crisis in the USA and other countries^1^. Existing treatments are inadequate and long-term success rates are poor^2^.

This clinical need has driven the search for new therapies, including modulation of brain regions implicated in addiction^1,3^. Trials of deep brain stimulation (DBS)^4^, transcranial magnetic stimulation (TMS)^5^ and surgical lesioning^6–10^ have targeted several different brain regions, with no consensus on the optimal target^4,5^. Given this ambiguity, a TMS device recently cleared by the U.S. Food and Drug Administration (FDA) for smoking cessation was designed to target multiple brain regions^11,12^. To better guide neuromodulation therapies, we need to know which brain regions are causally involved in addiction remission in human patients.

A unique source of information that can help answer this question is cases where brain damage such as a stroke results in remission of addiction in a patient^13–15^. These cases are valuable because they provide a causal link between therapeutic benefit and human neuroanatomy^16,17^. For example, lesions involving the insula are more likely to disrupt nicotine addiction than lesions that spare the insula^13^. However, lesions disrupting addiction have been reported outside the insula in many different brain locations, leaving localization unclear^13–15^.

Recently, it has been possible to link lesions in different brain locations to a common neuroanatomical substrate using the human connectome, a map of human brain connectivity^18,19^. When lesions result in therapeutic benefit, this approach can identify effective therapeutic targets^20,21^. For example, lesion locations that improve essential tremor are all connected to the exact spot in the thalamus that is an effective target for DBS^21^. In this study, we apply this method to lesions resulting in addiction remission.

## Results

**Fig. 1:**
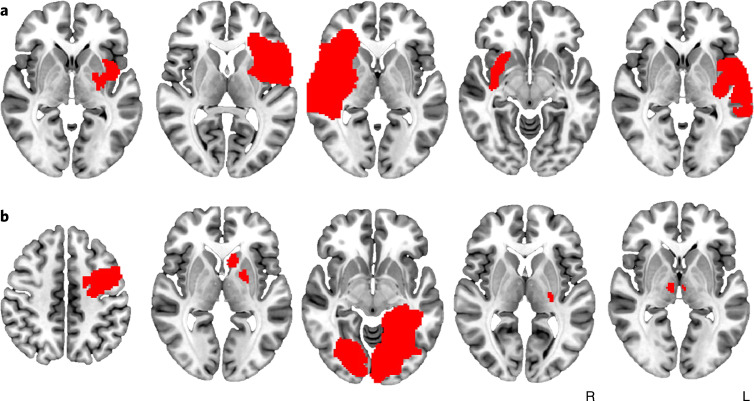
Lesions associated with addiction remission occur in many different brain locations. **a**,**b**, Some lesions associated with smoking addiction remission intersected the insula (**a**) but many others did not (**b**). Each slice represents a different patient and lesion locations are shown in red. Lesions from all 129 patients are shown in Supplementary Fig. 1.

Across two independent datasets (Supplementary Table 1), we identified 129 patients who were active daily nicotine smokers at the time of an acquired brain lesion: 69 patients (53%) continued smoking while 34 patients (26%) fulfilled the criteria for addiction remission (quit smoking without difficulty immediately after the lesion, did not relapse and reported absence of craving since quitting). On average (s.d.), patients smoked 23.1 (14.1) cigarettes per day; there was no difference between patients who remitted and those who did not quit (*P* = 0.98). Lesion locations were highly heterogeneous (Fig. 1 and Supplementary Fig. 1). Standard voxel-wise lesion-symptom mapping (VLSM) failed to identify any voxels significantly associated with addiction remission (*P* > 0.2), failed to show consistency between our 2 lesion cohorts (spatial similarity *r* = 0.05, permutation test *P* = 0.38) and failed to correlate with addiction remission across cohorts (*P* = 0.36) (Supplementary Fig. 2).

**Fig. 2:**
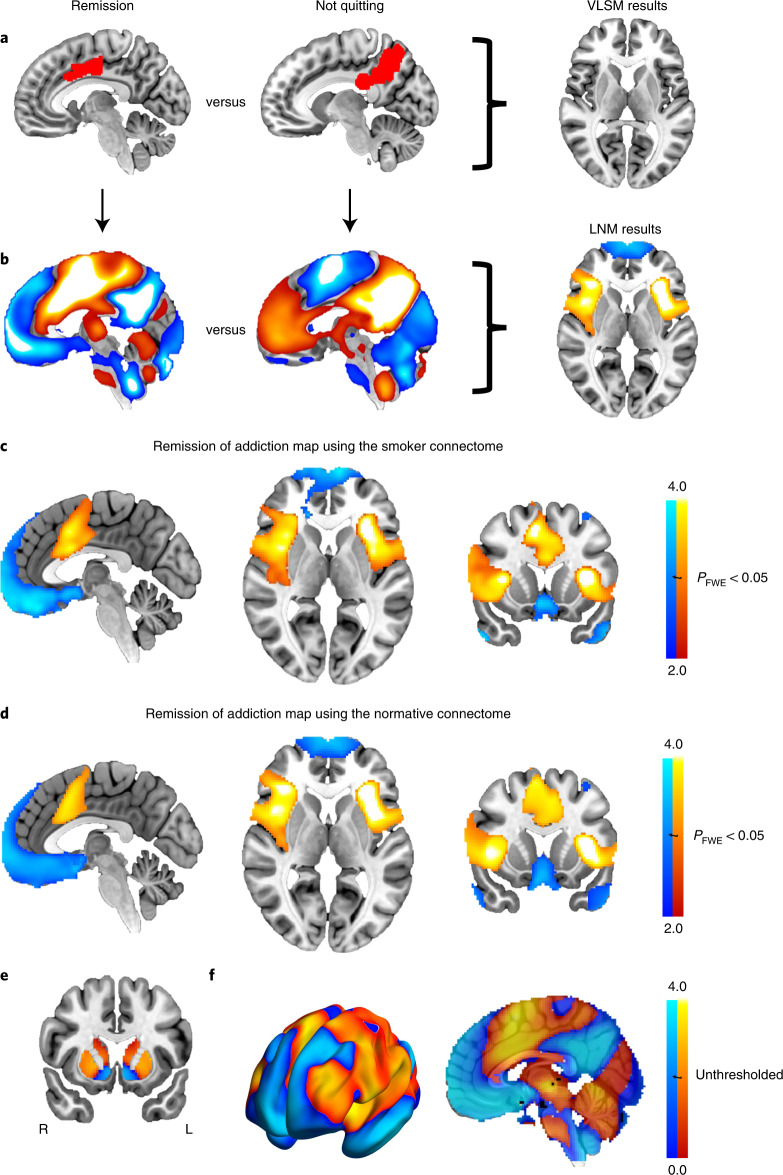
LNM of addiction remission. **a**, Lesion locations associated with remission of addiction to smoking (left, single example shown in red) and not quitting smoking (middle, single example shown in red) were compared using VLSM but with no significant findings (right). **b**, Next, the network of brain regions functionally connected to each lesion location was computed using LNM. Network maps are shown for the same two lesion examples from **a**. Positive functional connectivity values are shown in warm colors and negative functional connectivity values are shown in cool colors. Lesion network maps were statistically compared to identify connections significantly associated with smoking addiction remission (right, **b**). **c**–**e**, Lesion locations disrupting addiction showed a specific pattern of brain connectivity as computed using a smoker (**c**) or normative connectome (**d**,**e**). The maps derived using the normative connectome, which was used in all subsequent analyses, are displayed on the brain slices (**d**,**e**) and brain surface (**f**). This pattern included positive connectivity to the insula, dorsal cingulate and dorsolateral prefrontal cortex and negative connectivity to the medial prefrontal and temporal cortex (Supplementary Table 2). **c**,**d**, Maps were corrected for multiple comparisons using threshold-free cluster enhancement with default parameters in FSL (one-sided *P*_FWE_ < 0.05). **e**,**f**, Maps are shown unthresholded to better illustrate the dorsal/ventral dissociation in the striatum (**e**) and frontal cortex (**f**).

Given this heterogeneity in lesion locations, we next tested whether lesion locations disrupting addiction mapped to a connected brain circuit rather than one specific brain region. Connectivity patterns were compared between lesion locations resulting in smoking addiction remission and lesion locations from non-quitters (Fig. 2a,b). In contrast to our negative analysis of lesion location, we identified multiple significant differences in lesion connectivity (family-wise error-corrected *P* (*P*_FWE_) < 0.05; Fig. 2c,d and Supplementary Table 2). This result was independent of whether we used a connectome from active daily smokers (Supplementary Table 3) or a large normative connectome. We will refer to this map as our addiction remission network. Positive regions in this map showed stronger positive functional connectivity to lesion locations disrupting smoking addiction (versus lesion locations from non-quitters). Similarly, negative regions showed stronger negative functional connectivity to lesion locations disrupting smoking addiction (versus lesion locations from non-quitters) (Fig. 2c–f). As such, a lesion likely to lead to addiction remission would be positively connected to the cingulate and insula but negatively connected to the medial prefrontal cortex. Conversely, a lesion with the opposite connectivity profile would be the least likely to lead to addiction remission.

This connectivity pattern was reproducible when computed separately using our 2 independent lesion cohorts (spatial similarity *r* = 0.58, permutation test *P* = 0.03; Supplementary Fig. 3), was correlated with addiction remission across cohorts (*P* = 0.04) and was driven by similarity across lesions disrupting smoking addiction not similarity across lesions from non-quitters (U = 694, *P* < 0.001; Supplementary Fig. 4). The connectivity pattern was robust to different cutoffs for defining active daily smoking (Supplementary Fig. 5), adding lesion size as a covariate (Supplementary Fig. 6a,b), adding proportion of gray/white matter in the lesion mask as a covariate (Supplementary Fig. 6c), adding patient age as a covariate (Supplementary Fig. 6d), restricting our analysis to only strokes (Supplementary Fig. 6e), restricting our analysis to lesion masks defined with magnetic resonance imaging (MRI) (Supplementary Fig. 6f), using a connectome processed without global signal regression (Supplementary Fig. 7) or including insula damage as a covariate in a mediation analysis (Supplementary Fig. 8).

Our addiction remission network showed a striking dissociation in the striatum, changing from positive to negative at the boundary of the dorsal versus ventral striatum (striatal region × lesion group interaction *F*_(1,101)_ = 8.4, *P* = 0.005) (Fig. 2e). This dissociation was mirrored in the prefrontal cortex, with positive connectivity in the dorsolateral prefrontal cortex and negative connectivity in the ventromedial prefrontal cortex (Fig. 2f).

In the subset of patients who underwent neuropsychological testing, there were no significant differences between patients who remitted and those who did not quit smoking in intelligence quotient, executive function, working memory, verbal comprehension, mood or social introversion (Supplementary Table 4). An addiction remission map computed from just this subset of patients was nearly identical to the map generated using the full dataset (Supplementary Fig. 9).

To investigate anatomical connections that may underlie this functional network, we investigated damage to white matter tracts. Damage to 14 different tracts was associated with addiction remission (Supplementary Table 5). Two of these tracts (left fronto-insular tracts 1 and 2) survived correction for multiple comparisons. These tracts fell within 2 mm (1 voxel) of the whole brain peak of our addiction remission network derived using the functional connectome (*P*_FWE_ < 0.05; Supplementary Fig. 10a). Similarly, lesion locations resulting in addiction remission had higher white matter ‘disconnectome scores’ between nodes of our addiction remission network compared to lesion locations from non-quitters (*P* = 0.003; Supplementary Fig. 10b,c,d).

**Fig. 3:**
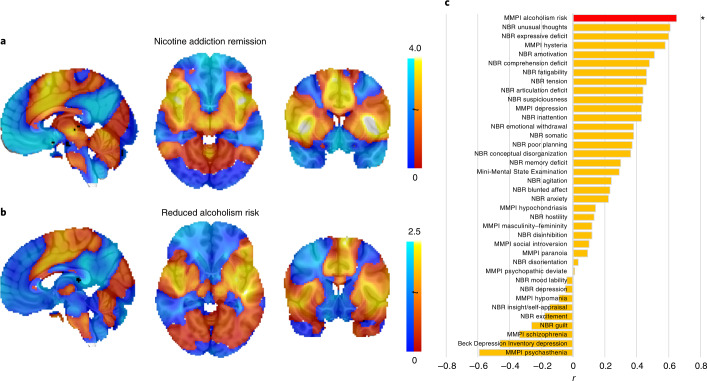
Generalizability of the smoking addiction remission network to alcoholism. **a**,**b**, The connectivity profile of lesions associated with addiction remission to smoking (**a**) was very similar to the connectivity profile of lesions associated with reduced alcoholism risk (**b**). Maps are shown unthresholded to facilitate visual comparison. **c**, This network similarity was specific to alcoholism risk since network maps generated for 37 other neuropsychological variables failed to match our addiction remission network. The alcoholism risk map was the only map that was more similar to our addiction remission network than expected by chance (permutation analysis, one-sided *P* = 0.04). NBR, neurobehavioral rating scale. *r*, map spatial correlation coefficient with the smoking addiction remission map. *P < 0.05, uncorrected.

To investigate generalizability, we studied 186 patients with lesions who completed an alcoholism risk assessment (Supplementary Table 6). Lesions associated with lower alcoholism risk showed similar connectivity to lesions that disrupted addiction to smoking (Fig. 3a,b; spatial *r* = 0.65, permutation test *P* = 0.04), even when controlling for smoking status (spatial *r* = 0.69, permutation test *P* = 0.04). This concordance between datasets was driven by network connectivity, not lesion location alone, since repeating the analysis using traditional VLSM failed to show similarity (spatial *r* = −0.15, *P* = 0.83). This concordance was also specific to addiction risk since maps generated for 10 other Minnesota Multiphasic Personality Inventory (MMPI) variables failed to match as well as the addiction risk map (*P* < 0.001) as did 27 maps generated using other neuropsychological variables (Fig. 3c). We also identified 3 case reports of lesions that disrupted addiction to substances other than nicotine and found network connectivity similar to that of lesions disrupting addiction to smoking (*P* < 0.05; Supplementary Fig. 11).

**Fig. 4:**
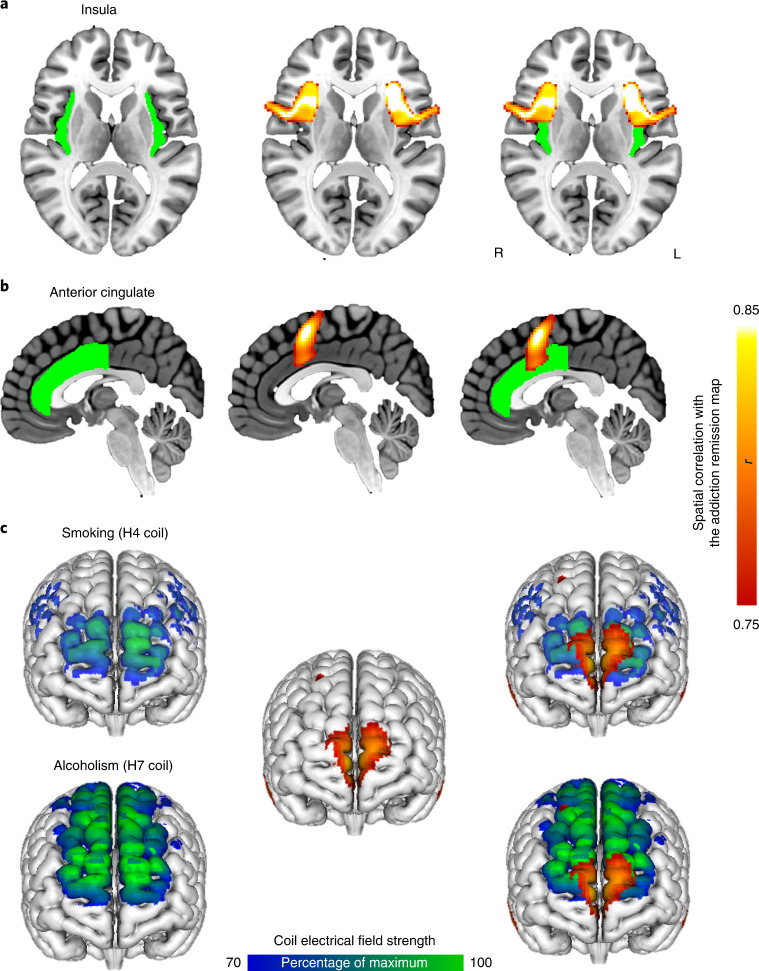
Refining neuroanatomical treatment targets for addiction. **a**–**c**, Neuroanatomical targets for treatment of addiction based on previous work included the insula (green, **a**), anterior cingulate (green, **b**) and frontal cortex (electric field models of the H4 and H7 TMS coils shown in blue-green, **c**). Voxels whose connectivity profile best matched our lesion-based addiction remission network included the left operculum/insula (red-yellow, **a**), paracingulate gyrus (red-yellow, **b**) and medial fronto-polar cortex (red-yellow, **c**). Therapeutic targets identified using LNM overlapped previous targets (right, **a**–**c**) but also provided testable hypotheses for how these targets might be refined or improved. Additional images for the electric field models of the H4 and H7 TMS coils (**c**) are shown in Supplementary Fig. 12.

Finally, we investigated which brain voxels have a connectivity profile that best matches the connectivity profile of lesion locations disrupting addiction. In theory, positive nodes in this map represent the ideal location to place a focal lesion to disrupt addiction, while negative nodes in this map represent the ideal location for excitatory brain stimulation, such as high-frequency TMS. Peak positive nodes were located in the left frontal opercular cortex adjacent to the left insula highlighted by prior lesion studies and in the paracingulate gyrus, just above the cingulate gyrus previously used as a surgical lesion target for addiction (Fig. 4 and Supplementary Table 7). The peak negative node was located in the medial fronto-polar cortex, overlapping the maximal electric field of the TMS coil recently approved by the FDA for smoking cessation and the TMS coil showing efficacy in the treatment of alcohol dependence in a proof-of-concept study (Fig. 4, Supplementary Fig. 12 and Supplementary Table 7).

## Discussion

There are several important findings from this study. First, lesions resulting in addiction remission occur in multiple different brain locations but are characterized by a specific pattern of connectivity to brain regions implicated in circuit-based models of addiction^3^. Second, the connectivity profile of lesions disrupting nicotine addiction is similar to the connectivity profile of lesions reducing the risk of alcoholism, suggesting a shared network for addiction across these substances of abuse. Finally, brain locations best matching the connectivity profile for addiction remission align with targets that have shown promise in treating addiction, suggesting potential therapeutic relevance of our results.

Studying brain lesions that cause specific neurological or psychiatric symptoms is a powerful method to localize human brain function, allowing for causal links between symptoms and neuroanatomy^16,17,19^. Previous studies found that lesions intersecting the insula are statistically more likely to result in addiction remission^13–15^ but other studies failed to replicate this finding or highlighted alternative brain regions, such as the basal ganglia^14,15^. Our findings help reconcile these results by showing that lesions associated with addiction remission map to a common brain circuit, not one specific brain region^3^. Our addiction remission network aligns well with neuroimaging abnormalities in patients with addiction and existing circuit-based models of addiction^1,3^. Our method complements this neuroimaging work by showing which neuroimaging correlates of addiction are linked to addiction remission based on brain lesions.

Lesion locations associated with addiction remission show positive functional connectivity to one set of brain regions (for example, insula, dorsal cingulate, dorsolateral prefrontal cortex) and negative functional connectivity to another set of regions (for example, ventral medial prefrontal cortex). These regions have been implicated in multiple different cognitive functions thought to be relevant for addiction and addiction remission^1,3^. The contrasting connectivity pattern to these two networks, mirrored also in the striatum, is consistent with clinical and preclinical data suggesting that an imbalance between networks may underlie vulnerability to addiction and relapse^22–24^. Our results may lend insight into which circuits are imbalanced and how this balance might be modulated for addiction remission. A lesion location connected to multiple brain regions may modulate multiple features of addiction simultaneously, correct imbalances (for example, decrease activity in the positively connected and increase activity in the negatively connected regions) and lead to addiction remission via subtle behavioral changes across multiple different domains. This view is supported by the lack of significant differences in any specific neuropsychological measurement between groups. However, it is possible that other behaviors that were not measured could be different between lesion groups.

Our analyses show concordance between results obtained using a functional and structural connectome; however, these approaches are fundamentally different and provide complementary information^19,25^. The functional connectome may be most useful in studying effects that map to polysynaptic brain networks and the structural connectome for effects that map to single white matter connections^25^. Further work is needed to determine how best to combine these approaches. Similar to previous work, we used a group connectome as an approximation of the connectivity of each individual patient, an approach that has certain advantages^19^. Our findings were nearly identical using a connectome from a group of smokers (*n* = 126) or a generic normative connectome (*n* = 1,000), which is consistent with previous work^18,26^. Future work may benefit from collecting diffusion MRI or connectivity imaging data in each patient with lesions.

Neuromodulation trials have shown some promise in treating addiction but have been limited by the fact that the optimal therapeutic target was unknown^5^. We hypothesize that the optimal lesion target for addiction is the brain region that best matches the connectivity profile of lesions disrupting addiction. These targets include the frontal operculum and paracingulate cortex, the latter being immediately adjacent to the anterior cingulate, the first lesion target used to treat addiction^6,7^. We also identified brain regions that showed the exact opposite (negative) connectivity profile of lesions associated with addiction remission, which we hypothesize is an optimal target for excitatory brain stimulation. The top negative peak is in the medial fronto-polar cortex, which has served as an optogenetic and TMS target to relieve addiction^5,27^. Interestingly, the TMS coil recently cleared by the FDA to treat smoking cessation was designed to target the insula and dorsolateral prefrontal cortex but the peak electric field intensity was actually in the fronto-polar cortex, overlapping our lesion-based target for addiction remission^11,12^. Similarly, a different TMS coil showing efficacy in the treatment of alcohol dependence, designed to target the medial prefrontal and anterior cingulate cortices, also overlaps our hypothesized TMS target^28^. Both of these trials used 10-Hz repetitive TMS thought to exert an excitatory effect on the underlying brain tissue, which is also consistent with our hypothesis. Finally, our results implicate a network of multiple regions, not just a single region, in addiction remission. Treatments designed to excite and inhibit different regions simultaneously, such as multifocal electrode arrays, may prove useful^29^.

Strengths of our study include replication of results across three independent datasets, use of causal lesion data from human patients and the generation of testable hypotheses and therapeutic targets for use in future work. There are also several limitations. First, our addiction remission network is derived from nicotine smokers, potentially limiting generalizability to other SUDs. However, this addiction remission network was consistent with lesions that reduced the risk of alcohol addiction, case reports of lesions disrupting addiction to other substances and TMS coil models for both smoking and alcoholism, which is consistent with a shared network for addiction across drugs of abuse^1,3,22^. However, it should be noted that personality factors that predispose to alcoholism, as measured with the MacAndrew Alcoholism Scale (MAC), are not equivalent to actually having an addiction to alcohol. Second, exposure to cigarettes after discharge from hospital, the ability to conduct daily function and socioeconomic indicators, such as family support, income and location of residence or residential status were not assessed, although this would add noise to the data and bias us against the current findings. Third, neuropsychological scores were only available from a subset of patients. However, our addiction remission map was almost identical when calculated using this subset, suggesting that the sample was representative of the full dataset. Finally, while our study aligns with previous neuromodulation targets for addiction and identifies specific coordinates designed to optimize these effects, these targets are to be prospectively tested in randomized clinical trials. Similarly, the side effect profile of treatments directly targeting the nodes of our addiction remission circuit is to be assessed since this could differ from existing data on cingulotomy or medial prefrontal TMS^6–8,11,30^. Whether noninvasive brain stimulation, such as TMS, can provide a lasting change to the behavior is to be determined.

In summary, heterogeneous lesion locations disrupting addiction are characterized by a specific pattern of connectivity and hubs that best capture this pattern provide data-driven, testable targets for therapeutic neuromodulation. However, side effects potentially associated with these targets are unknown and must be characterized in detail.

## Methods

For full details of each analysis, please see the supplementary information.

### Smoker lesion cohorts

Two cohorts of patients who were active daily nicotine cigarette smokers at the time of focal brain damage were analyzed retrospectively: one cohort from the University of Iowa^13^ and a second cohort from the University of Rochester^15^. Because previous publications using these cohorts used qualitative review of structural brain scans^13,17^, we returned to the original computed tomography/MRI data to generate precise outlines of each lesion location in the atlas space. Cases without clearly identifiable lesions or diffuse lesions were excluded. Altogether 129 brain lesions were available for analysis (*n* = 67 and *n* = 62 in the Iowa and Rochester datasets, respectively). The outcome of smoking behavior was assessed at the time of the original study in person or via telephone (only in person in the Rochester cohort). Disruption of smoking addiction was defined using previously established criteria^13,15^: the patient reported quitting smoking less than a day after their brain lesion, rated difficulty of quitting 1 or 2 on a scale of 1–7, reported not starting smoking again since their brain injury and reported that they felt no urge to smoke since quitting. For simplicity, we use the term ‘addiction remission’ to refer to patients meeting these criteria.

### VLSM

Brain lesion locations were manually traced and transferred to the MNI space. VLSM was used to test for associations between lesion location and clinical outcome using the NiiStat software v1.0.20191216 (https://github.com/neurolabusc/NiiStat). Study site and lesion size were included as covariates and lesioned voxels associated with smoking addiction remission (*n* = 34, 26%) versus not quitting (*n* = 69, 53%) were identified. Patients who quit smoking but did not meet criteria for addiction remission (for example, continued to crave cigarettes) were included in the model to maximize spatial coverage of the lesions and statistical power. Reproducibility was assessed by analyzing each cohort separately, testing for similarity across cohorts and determining whether addiction remission in one cohort could be determined using results from the other cohort.

### Lesion network mapping

A recently developed method, termed lesion network mapping (LNM), was used to investigate functional connectivity between each lesion location and all other brain voxels^18,19^. To ensure that the results were independent of the connectome, LNM analyses were conducted separately using two different connectomes: a connectome derived from 126 current smokers and another derived from 1,000 healthy volunteers.

Briefly, lesion locations were used as seed regions for resting-state functional connectivity MRI analysis using the connectome data. One lesion mask for each patient (*n* = 129) was generated in the MNI space and used as a seed region for connectivity analyses. Each patient’s lesion mask was used as a single seed. Seed-based functional connectivity between the lesion mask and the rest of the brain was computed using resting-state functional connectivity data from individuals included in the connectome, resulting in 126 or 1,000 (according to the number of individuals included in the connectome) functional connectivity maps per seed. The maps in the connectome were combined to create a single connectivity map per seed, which we call a ‘lesion network’ (one for each patient). These 129 lesion networks were then utilized in a single general linear model with study site as a covariate to identify connections associated with smoking addiction remission (below). Because the normative connectome is larger, showed better split-half reliability (Supplementary Fig. 7) and is more generalizable, we used this connectome for subsequent analyses; however, the addiction remission network was nearly identical using either the smoker or normative connectome.

### Connectivity profile of addiction remission

At each voxel, a general linear model was constructed to relate lesion connectivity to clinical outcome (addiction remission, quitting without remission, not quitting). Study site was included as a covariate and connections associated with addiction remission versus not quitting were identified. The analysis was conducted across the entire brain and statistical significance was set at *P*_FWE_ < 0.05 using threshold-free cluster enhancement implemented in FSL v.6.0 (https://fsl.fmrib.ox.ac.uk/fsl). Reproducibility across lesion cohorts was assessed using the same criteria described above for VLSM. Robustness was assessed by using different cutoffs for defining active daily smoking, adding lesion size as a covariate, adding gray/white matter proportion in the lesion mask as a covariate, adding patient age as a covariate, limiting the analysis to only stroke lesions, limiting the analysis to lesion masks defined with MRI, comparing the results between the two connectomes, using a connectome processed without global signal regression and testing whether our connectivity findings were mediated by damage to the insula. To test whether differences in addiction remission were associated with other cognitive/behavioral effects, we investigated neuropsychological scores in a subsample of patients who underwent dedicated testing after the occurrence of the lesion (*n* = 57).

Given previous work implicating the dorsal (dorsal caudate and dorsal putamen) versus ventral striatum (nucleus accumbens, ventral caudate and ventral putamen) in addiction^3^, we created two striatal regions of interest (ROIs), as described in detail elsewhere^31^. Functional connectivity between each lesion location and these striatal ROIs was calculated using the normative human connectome data. The resulting correlation coefficients were *z*-transformed and differences in lesion connectivity to the ventral versus dorsal striatum was investigated using a multivariate analysis of variance (ROI × group interaction).

### Structural connectivity profile of addiction remission

Lesion locations from each individual were mapped onto tractography reconstructions of 68 white matter pathways distributed with BCBtoolkit^32^. Damage to white matter tracts was quantified using the Tractotron tool in BCBtoolkit^33^. For each white matter tract, a general linear model using PALM^34^ was constructed to relate tract damage to clinical outcome (addiction remission, quitting without remission, not quitting). Study site and lesion size were included as covariates and damage to white matter tracts associated with addiction remission versus not quitting were identified. We controlled for multiple comparisons across the 68 white matter tracts using PALM analogous to our voxel-wise analyses^34^; statistical significance was set at *P*_FWE_ < 0.05 (one-tailed, as only considering addiction remission > not quitting).

### Associations between lesion structural and functional connectivity

Structural disconnection maps were calculated using the Disconnectome tool in BCBtoolkit^33^. As in previous work with this toolkit^35^, we utilized diffusion-weighted imaging data from 178 healthy controls^36^, identified white matter fibers passing through each lesion location^37,38^ and then transformed these fiber maps into binarized visitation maps in the MNI152 space and summed these maps across individuals^39,40^. This process resulted in a single ‘structural disconnection map’ for each lesion and reflects the probability of structural disconnection between the lesion location and each brain voxel^40^. To test for associations between these structural disconnection maps and our addiction remission network (derived from the functional connectome), we summed the voxels from each structural disconnection map that intersected positive regions in our addiction remission network (Fig. 2d and Supplementary Table 2). This generated a single structural ‘disconnectome score’ for each lesion. A general linear model using PALM^34^ was constructed to relate the structural disconnectome score to clinical outcome (addiction remission, quitting without remission, not quitting) and the contrast of interest was addiction remission versus not quitting. Study site and lesion size were included as covariates. Statistical significance was set at *P* < 0.05 (one-tailed hypothesis testing was used because the hypothesis was directional: addiction remission > not quitting).

### Generalizability across SUDs

Brain lesions from individuals with penetrating focal brain injury, part of the Vietnam head injury study^41^, were analyzed retrospectively (*n* = 186). Although we could not assess addiction remission in this dataset (information on substance use before brain injury was not collected), we could study addiction risk using the MAC, a subscale of the MMPI that measures personality characteristics that predispose to alcoholism. Connectivity with each lesion location was computed as described earlier. Connections correlated with MAC score were identified, yielding a map of connections associated with alcoholism risk. The similarity of this map with our smoking addiction remission map was calculated using spatial correlation and verified using a permutation analysis^42^. To test for specificity to addiction risk, we repeated our analyses using the 10 other domains of MMPI and 27 other behavioral variables available in this dataset.

Finally, a literature search was conducted to identify case reports of lesions resulting in remission of non-nicotine SUDs. Lesion location and connectivity in each case were computed and compared to our smoking addiction remission map.

### Neuroanatomical treatment targets

To determine which brain voxels had a connectivity profile that best matched the profile for lesion-induced addiction remission, a connectivity map for each brain voxel was computed and compared to our smoking addiction remission map using spatial correlation. This generated a voxel-wise map where the intensity of each voxel reflected the match to our addiction remission map. This map was thresholded at *r* > 0.75 and *r* < −0.75 to show only the peak findings. Results were overlaid on existing lesion targets for addiction, such as the anterior cingulate and insula (ROIs from the Harvard–Oxford atlas), and existing TMS targets for smoking and alcoholism (BrainsWay TMS H4 and H7 coil electric field models, respectively)^6–8,11–13,28^. The H4 coil was recently approved by the FDA for short-term smoking cessation (https://www.accessdata.fda.gov/cdrh_docs/pdf20/K200957.pdf).

The study was approved by the local institutional review boards (Beth Israel Deaconess Medical Center (no. 2018P000128) and Brigham and Women’s Hospital (no. 2020P002987)); all individuals provided written informed consent as part of the original study they were enrolled in.

### Reporting summary

Further information on research design is available in the Nature Research Reporting Summary linked to this article.

## Online content

Any methods, additional references, Nature Research reporting summaries, source data, extended data, supplementary information, acknowledgements, peer review information; details of author contributions and competing interests; and statements of data and code availability are available at 10.1038/s41591-022-01834-y.

## Supplementary information

Supplementary Information Supplementary Methods, Figs. 1–12 and Tables 1–7.

Reporting Summary

## Data Availability

De-identified lesion masks in the MNI atlas space from our two primary datasets (Iowa and Rochester cohorts) are available from Harvard Dataverse (10.7910/DVN/8BHHRS). Clinical and behavioral data from the patients with lesions is available upon request, subject to the policies and procedures of the institution where each dataset was collected. Data requests should be sent to the corresponding authors.

## Code availability

The code for the lesion connectivity analysis is freely available in the Lead-DBS toolbox (www.lead-dbs.org). We have made a fully preprocessed version of our 1,000 individual functional connectome publicly available (10.7910/DVN/ILXIKS), which was created using the Brain Genomics Superstruct Project data (10.7910/DVN/25833), along with all of the code and parameters used to process the data (10.5281/zenodo.4905738).
