# Supplementary material for: Brain lesions disrupting addiction map to a common human brain circuit
Source: Nat Med. 2022 Jun 13;28(6):1249–55. doi: 10.1038/s41591-022-01834-y (PMC9205767; doi:10.1038/s41591-022-01834-y)
Supplement: Supplementary file 1 — Supplementary Methods, Figs. 1–12 and Tables 1–7. [file 41591_2022_1834_MOESM1_ESM.pdf]

nature portfolio

https://doi.org/10.1038/s41591-022-01834-y

Supplementary information

Brain lesions disrupting addiction map to a common human brain circuit

In the format provided by the
authors and unedited

Supplementary Material

Brain lesions disrupting addiction map to a common human brain circuit

Juho Joutsa, MD, PhD, Khaled Moussawi, MD, PhD, Shan H. Siddiqi, MD, Amir Abdolahi, PhD, MPH, William Drew, BA, Alexander L. Cohen, MD, PhD, Thomas J. Ross, PhD, Harshawardhan U. Deshpande, PhD, Henry Z. Wang, MD, PhD, Joel Bruss, BA, Elliot A. Stein, PhD, Nora D. Volkow, MD, Jordan H. Grafman, PhD, Edwin van Wijngaarden, PhD, Aaron D. Boes, MD, PhD, Michael D. Fox, MD, PhD

Drs. Joutsa, Moussawi and Siddiqi contributed equally to this article.

Table of contents

Supplementary methods
Figure S1. Smoker cohort lesions
Figure S2. Lesion locations and voxel-lesion symptom mapping (VLSM) results.
Figure S3. Addiction remission network maps are reproducible when computed in each dataset independently.
Figure S4. Lesion network overlap results.
Figure S5. Results are robust to different cut-offs for active daily smoking
Figure S6. Addiction remission network results are robust to methodological choices.
Figure S7. Addiction remission network results are robust to the connectome
Figure S8. Mediation analysis
Figure S9. Subsamples with neuropsychological assessment are representative for the full sample
Figure S10. Structural connections related to addiction remission
Figure S11. Generalizability of findings to other substance use disorders
Figure S12. Brainsway coil electric field (E-field) models
Table S1. Demographical data for smoker lesion datasets.
Table S2. Significant clusters in the addiction remission network map.
Table S3. Demographical data for the smoker connectome subjects
Table S4. Neuropsychological test scores
Table S5. White matter tracts associated with addiction remission
Table S6. Demographical data for the Vietnam Head Injury Study
Table S7. Regions whose connectivity profile best matches the addiction remission network map.

Supplementary methods

Overview:

We leveraged data from two existing cohorts in which information on smoking addiction was collected following a focal brain lesion (**Table S1**).[13,15] Prior publications using these cohorts tested whether lesions intersecting the insula (based on qualitative review of structural brain scans) were associated with disruption of addiction.[13,15] For the current study, we returned to the original structural brain scans to generate precise outlines of each lesion location in atlas space. We then used a new technique termed lesion network mapping to test a new hypothesis, namely whether lesions associated with disruption of addiction map to a specific brain circuit [18,19]. This analysis was not possible when the datasets were originally collected as it requires a wiring diagram of the human brain called the human connectome, which has only recently become available.[18,19] The human connectome is generated using resting state fMRI data from a large cohort of healthy volunteers (n=1,000).[43,44] In lesion network mapping, the connectome is used to approximate connectivity with the lesion location at the time of the lesion. As such, lesion network mapping does not require connectivity imaging of the patients themselves. This is an important advantage, as the technique can be used with previously collected lesion cohorts in which only structural brain scans are available.[18,19] It is worth noting that even if resting state fMRI data had been collected in these patients after the lesion, it would not be helpful for this analysis, as connectivity with lesion locations can't be computed after the lesioned tissue is lost.

Iowa Lesion Cohort

Active daily smoking was defined as smoking more than five cigarettes per day for more than two years at the time of lesion onset.[13] From the original 69 patients in the University of Iowa cohort,[13] two patients were excluded (one because the original scan was not found and one without a clearly identifiable focal brain lesion). Of these 67 patients, lesions from 47 patients were manually traced onto a template brain using the MRI or CT scan as a guide.[45,46] Because lesion tracing methods at the University of Iowa were changed in 2006, the other 20 patients had their lesions manually traced on native T1-weighted scans with FSL and then transformed to the MNI152 atlas using nonlinear registration and lesion masking techniques available in ANTs software.[47] Lesion masking techniques included enantiomorphic normalization, which replaces the lesion volume with the voxel intensities from its non-damaged homologue, and applying a cost function to mask to the lesion volume for bilateral lesions.[48] The anatomical accuracy of all lesion tracings was reviewed in both native and MNI space and edited as needed by a neurologist (A.D.B.) blinded to clinical outcome data.

Rochester Lesion Cohort

Active daily smoking was defined as smoking at least one cigarette per day and at least 100 in their life-time at the time of lesion onset.[15] From the 156 patients in the University of Rochester cohort,[15] brain imaging data was available for 66 patients. Four patients were excluded because there was not a well-defined focal brain lesion (three had extensive hemorrhages and one lacked a clearly identifiable focal brain lesion). The remaining 62 brain lesions were manually traced on the MNI template based on the original brain MRI or CT by a neurologist (K.M.) blinded to clinical outcome data.

Both Cohorts:

Disruption of smoking addiction was defined identically in both cohorts using previously established criteria:[13,15] the patient reported quitting smoking less than a day after their brain lesion, rated difficulty of quitting 1 or 2 on a scale of 1-7, reported not starting smoking again since their brain injury, and reported that they felt no urge to smoke since quitting. We will continue to use the term "smoking addiction" as it has been specifically defined for these cohorts, however updated terms including "nicotine dependence" or "substance/tobacco use disorder" can be considered analogous. For simplicity, we will also use the term "addiction remission" to refer to patients meeting the above criteria "disruption of smoking addiction".

Voxel-based lesion symptom mapping

VLSM analyses were conducted using standard methods and NiiStat software (https://github.com/neurolabusc/NiiStat).[17] The analysis was conducted voxel-by-voxel across the whole brain. The contrast of interest was addiction remission vs. not quitting. Positive values in this VLSM map identify voxels more likely to be lesioned in addiction remission cases while negative values identify voxels more likely to be lesioned in non-quitters. Statistical significance was set at $p<0.05$ using

Freedman-Lane permutation with 2000 permutations. Voxels not affected by any of the lesions were excluded from the analyses. Any significant findings were confirmed by repeating the analysis but excluding voxels affected in < 5% of the cases (n < 7). To assess for reproducibility across our two smoking datasets, the main analysis was repeated on each dataset individually, generating two independent VLSM maps for addiction remission (**Fig S2C**). We tested for similarity between these two maps using spatial correlation across all non-zero voxels, as in prior work from our group. Spatial correlation was selected to avoid any potential bias associated with arbitrary thresholds or weighting of certain regions, because it provides a simple metric of how much variance in one map matches another, and because if was used in our prior work.[49-51] This spatial correlation value was compared to a null distribution derived from randomly permuting clinical outcome and repeating the analysis (1000 iterations). We also tested whether clinical outcome in one dataset could be determined using the VLSM results from the other independent dataset. For this analysis, we computed the intersection of each lesion location with the VLSM map derived from the other dataset, summing the VLSM voxel values within the lesion mask. This generates a single number for each lesion that reflects the probability of addiction remission based on the VLSM results from the other dataset. Values for the addiction remission lesions were compared to the values from non-quitters using an independent sample two-tailed t-test.

Lesion network mapping

To ensure that the results are independent of the subjects included in the connectome, lesion network mapping was conducted separately using two different connectomes: a connectome derived from current smokers (n=126) and connectome derived from healthy volunteers (n=1,000) using practically identical methodology. Detailed connectome robustness analyses comparing the two connectomes are described in "Robustness of smoker versus normative connectome to split-half replication".

Lesion network mapping was used to investigate functional connectivity between each lesion location and all other brain voxels using the same methods described extensively in prior work.[18,19,44] Briefly, lesion locations were used as seed regions for resting state functional connectivity MRI (rs-fcMRI) analysis using the two connectomes in separately. Pearson correlation maps of connectivity were transformed to a normal distribution using Fisher r-to-z transformation and averaged across all subjects in the connectome. This method identifies the network of brain regions functionally connected to each lesion location without the need for functional imaging data from the patients themselves.

Smoker connectome used for lesion network mapping

For the smoker connectome, participants were required to be right-handed, 18 to 60 years of age, free of active drug or alcohol abuse/dependence (other than nicotine), report no current psychiatric or neurological disorders, and have no contraindications for magnetic resonance imaging (MRI). Written informed consent was obtained in accordance with the National Institute on Drug Abuse Intramural Research Program Institutional Review Board. Data from a total of 126 participants recruited over four separate study protocols were included in the analyses. The demographics for the participants are summarized in Table S3. MRI data acquisition parameters: Whole-brain echo planar images were acquired on a 3T Siemens Allegra (n=81), Siemens Trio (n=35) and a Siemens Prisma scanner (n = 10). High-resolution oblique–axial T1-weighted structural images were acquired using a 3D magnetization-prepared rapid gradient-echo (MPRAGE) sequence (repetition time (TR)=2000 ms (n=45), 2500 ms (n=81); echo time (TE)=3.5 ms (n=45), 4.38 ms (n=81); inversion time (TI) = 900 ms (n=45), 1000 ms (n=81), flip angle (FA)= 9° (n=45), 8° (n=81), voxel size=1 mm3). The resting state fMRI data were collected as oblique axial slices, using a T2*-weighted, single-shot gradient echo, echo planar imaging sequence sensitive to blood oxygenation level-dependent (BOLD) effects (TR=2000 ms; TE=27 ms; FA= 80° (n=69), 78° (n=45), 77° (n=12); slice thickness= 4 mm (n=75), 3.5 mm (n=51); field of view 220 × 220 mm2; image matrix 64 × 64; run length=180 frames/6 minutes). Structural data were processed using FreeSurfer version 6.0.1 to generate cortical surface models and subcortical segmentations that were then used for the registration and regression steps of the functional data preprocessing below.[52,53]

Resting state functional data were processed with Dr. Thomas Yeo's Computational Brain Imaging Group's fMRI preprocessing pipeline (https://github.com/ThomasYeoLab/Standalone_CBIG_fMRI_Preproc2016) with the aid of wrapper scripts that allow the application of this pipeline to BIDS-formatted data (https://github.com/bchcohenlab/BIDS_to_CBIG_fMRI_Preproc2016) in similar fashion to the primary connectome data described in the main text. Preprocessing included the following steps: 1) removal of the first 4 frames/TRs of data, 2) slice time correction with FSL,[54,55] 3) motion correction using FSL's mcflirt, and motion outliers were identified using fsl_motion_outliers at thresholds of framewise displacement > 0.2mm or DVARS > 50, with segments less than 5 frames also

being excluded [56], 4) alignment of structural and functional images using boundary-based registration[57] from the FsFast software package (http://surfer.nmr.mgh.harvard.edu/fswiki/FsFast), and 5) nuisance removal via regression of whole brain/global signal, twelve motion correction parameters, averaged ventricular signal, averaged white matter signal, as well as their temporal derivatives (18 regressors in total). The flagged outlier volumes were ignored during the regression procedure. 6) The data were interpolated across censored frames using least squares spectral estimation of the values at censored frames,[58] and 7) finally, a band-pass filter ($0.009 \leq f \leq 0.08$ Hz) was applied. The preprocessed data was then projected to the 2mm version of the MNI152 NLIN 6th generation atlas packaged with FSL using mri_vol2vol and smoothed with a FWHM of 6mm.

Functional connectivity maps were then created using AFNI (https://afni.nimh.nih.gov/ version 20.2.06): AFNI's 3dmaskave was used to extract the averaged time series from the residual files for each of the regions of interest (ROIs) for every participant. These time series were then used as seed regions in a whole-brain seed-based functional connectivity (FC) analysis using the 3dDeconvolve command. The correlation coefficients were then converted to z-scores using the Fisher's r-to-Z transform. Finally, for each of the seed ROIs, a group t-statistic map versus zero was calculated over the 126 participants using AFNI's 3dttest++.

Normative connectome used for lesion network mapping

The normative resting state functional connectivity data used in this study is derived from the Brain Genomics Superstruct Project (GSP) dataset of 1,570 healthy young adult participants.[43] Versions of this dataset have been used for functional connectivity analysis for over a decade [59,60] and prior lesion network analyses from our group.[19,61] We have made a fully pre-processed version of this connectome publicly available [https://doi.org/10.7910/DVN/ILXIKS], along with all of the code and parameters used to process the data [https://doi.org/10.5281/zenodo.4905738].

The GSP data was acquired on matched Siemens 3T MAGNETOM Tim Trio MRI systems (Erlangen, Germany) at Harvard University and Massachusetts General Hospital using the vendor-supplied 12-channel phased-array head coil. Structural data included a high-resolution (1.2 mm isotropic) multi-echo T1-weighted magnetization-prepared gradient-echo image (multi-echo MPRAGE). Functional imaging data were acquired using a gradient-echo echo-planar imaging (EPI) sequence sensitive to blood oxygenation level-dependent (BOLD) contrast. BOLD runs consisted of 47 interleaved slices (foot—head; 1, 3, 5 … 45, 47, 2, 4, 6 …, 44, 46). One hundred and twenty-four measurements were collected for each BOLD run (TR=3000 msec; 4 initial TRs collected to allow for T1-stablization and 120 valid measurements). During BOLD data collection participants were instructed to remain still, stay awake, and keep their eyes open while blinking normally. One or two BOLD runs were acquired per subject (72.6% of sessions included two runs). Sequences, parameters, and instructions were unchanged throughout the collection process. However, not all subjects were acquired on the same scanner, as five different scanners were used. In addition, during the scanning period, the scanner console changed from B13 to B15 to B17. The scanner (Scanner_Bin) and console version (Console) for each imaging session are available within the CSV files included in the original data release (GSP_list_140630.csv and GSP_retest_140630.csv). GSP subjects included in this connectome were chosen based on a combined 3-way score of normalized DVARS, normalized Entropy Focused Criterion (EFC), and normalized Framewise Displacement (FD) values generated from mriqc (https://mriqc.readthedocs.io/en/stable/) consistent with the goal of minimizing the effects of motion without "scrubbing".[62] The 1000 GSP subjects with the best combined scores were selected for connectome inclusion. Additionally, if a subject contains 2 resting-state BOLD runs, then the 2 motion quality values are averaged to produce a single value. Anatomical surfaces were created with FreeSurfer v4.5.

Functional preprocessing was then performed with a modified version of Thomas Yeo's Computational Brain Imaging Group (CBIG) fMRI preprocessing pipeline to generate the BOLD runs used in the connectome that allows for separate subcortical, cerebellar, and cortical spatial smoothing, as was done in,[59] but is not currently enabled in the latest versions of CBIG Pipeline. Our complete code and wrapper script to allow application of the CBIG pipeline to BIDS formatted data is publicly available at [https://doi.org/10.5281/zenodo.4905738]. The CBIG pipeline allows for a configurable sequence of steps for functional connectivity preprocessing and the exact parameters used here are available in our 'config file' online [https://github.com/bchcohenlab/BIDS_to_CBIG_fMRI_Preproc2016/blob/1.0.0/configs/Legacy_GSP.config]. Briefly, preprocessing included the following steps: 1) removal of the first 4 frames/TRs of data, 2) slice time correction with FSL,[54,55] 3) motion correction using FSL's mcflirt, and motion outliers were identified using fsl_motion_outliers at thresholds of framewise displacement > 0.2mm or DVARS > 50, with segments less than 5 frames also being excluded [56], 4) alignment of freesufer surfaces and structural and functional images using boundary-based registration[57] from the FsFast software package (http://surfer.nmr.mgh.harvard.edu/fswiki/FsFast), and 5) nuisance removal via regression of whole brain/global signal, twelve motion correction parameters, averaged ventricular signal, averaged white matter signal, as well as their temporal derivatives (18 regressors in total). The flagged outlier volumes were ignored during the regression procedure. 6)

Next, a band-pass filter ($0.0001 \leq f \leq 0.08$ Hz) was applied. 7) Finally, the preprocessed data was then projected to the 2mm version of the MNI152 NLIN 6th generation atlas packaged with FSL using mri_vol2vol and smoothed with a FWHM of 7mm. Data from multiple runs, where available, was then concatenated.

Connectivity profile of addiction remission / addiction remission network

At each voxel, a general linear model was constructed to relate lesion connectivity to clinical outcome (addiction remission, quitting without remission, not quitting). Study site was included as a covariate and connections associated with addiction remission vs. not quitting were identified. The analysis was conducted across the entire brain and statistical significance was set at FWE-corrected $p<0.05$ using threshold-free cluster enhancement (tfce) implemented in FSL randomise.[34]

Robustness of Smoker versus Normative Connectome to Split-Half Replication

Our primary results were nearly identical using the smoker versus the normative connectome. To guide our decision of which connectome to use in our many subsequent analyses (e.g. comparison with lesions associated with reduced alcoholism risk) we tested the robustness of each connectome to split-half replication. The robustness of the connectomes was assessed by splitting the connectome into two randomly selected halves (n=500 for the normative connectome and n=63 for the smoker connectome) and obtaining two randomly selected smaller samples from the normative connectome matching the number of subjects in split halves of the smoker connectome (n=63). We then computed the spatial correlation coefficient (r) between the resulting lesion connectivity maps within each connectome pair. The test-retest reliability within each connectome was then investigated by comparing the explained variance (r^2) between the connectomes (**Fig. S7**).

Reproducibility of the addiction remission network

Reproducibility was assessed by repeating the above analysis independently in the Iowa and Rochester lesion datasets, identical to the reproducibility testing performed for the VLSM analysis (**Fig S3**). The spatial correlation across non-zero voxels in each map was computed. This correlation value was compared to a null distribution derived from randomly permuting group labels (1000 iterations). To test for clinical predictive value, within each dataset, spatial correlation was computed between each subject's lesion connectivity and the lesion network map generated from the other dataset. These spatial correlations were compared between addiction remission vs. not quitting across both datasets using independent samples t-test (two-tailed). To demonstrate that our findings are specific for connectivity, the same reproducibility analyses were conducted using the lesion network maps and the VLSM maps.

Determining which group drives the addiction remission network

To investigate which group drove the connectivity difference, we conducted two analyses. First, we determined the similarity of the connectivity maps within each group by computing the spatial correlation between each individual map and the average map for that group. The resulting correlation coefficients were compared between groups using Mann-Whitney U-test. Second, to investigate commonly connected regions within each group, we conducted a lesion network overlap analysis (**Fig S4**). Individual 'lesion networks' were created from the voxelwise connectivity maps, which were thresholded with $|t| \geq 5$ corresponding to uncorrected $P<10^{-6}$ and whole brain voxel-level FWE-corrected $P<0.05$. The thresholded maps were binarized then overlaid to show brain regions connected to the greatest number of lesion locations. This analysis was performed separately for positive and negative connectivity and for lesions associated with addiction remission and not quitting. Higher lesion network overlap indicates greater consistency in the lesion networks.

Robustness of the addiction remission network to methodological choices

To ensure our results were robust to methodological choices, we repeated our analysis multiple times varying our inclusion criteria, covariates, cutoffs, and even our connectome. For all analyses, similarity to the original addiction remission network was assessed through spatial correlation.

1. First, we repeated our analysis varying our cutoff for classifying a subject as an "active daily smoker" (**Fig S5**). Note that this had been defined differently in the original studies of the Iowa and Rochester cohorts. To exclude the possibility that these different definitions impacted our results, we recomputed the addiction remission map after excluding eight patients

from the Rochester cohort who smoked less than five cigarettes per day (n = 121). We also repeated our analysis including only patients who smoked at least 20 cigarettes per day (n = 86).

2. Second, we repeated our analysis including lesion size (**Fig S6B**), proportion of gray/white matter in the lesion mask (**Fig S6C**) and age (**Fig S6D**) as a covariate. Although lesion size is usually included as a covariate in traditional lesion analyses,[17] it is not usually included in lesion network mapping analyses.[18,44,63-68] However, we sought to verify that the results were not influenced by lesion size and to make our lesion network mapping results directly comparable to the traditional VLSM results. Proportion of gray/white matter in the lesion masks was defined by extracting the number of voxels falling in the FSL gray and white matter tissue prior maps (threshold >0.5 for both) and calculated the difference of their proportions from the total number of voxels in the lesion, resulting in values from -1 (fully white matter lesion) to +1 (fully gray matter lesion).

3. Third, to verify that our results are not dependent on the lesion etiology or imaging modality, we replicated the main analysis in strokes only, which constituted 118/129 of our lesions (**Fig S6E**), and lesion masks defined with MRI only (**Fig S6F**).

4. Fourth, we run our analysis using a connectome derived from active daily smokers (n=126) and our standard connectome derived from healthy (non-smoking) individuals (n = 1,000) (**Fig. 2C-D, Fig S7**).

5. Fifth, to assess the effect of global signal regression on our results, we repeated our main analysis using a connectome processed without global signal regression.[69] Specifically, we processed the first 100 subjects of the 1,000 subject healthy volunteer connectome using aCompCor and the Conn Toolbox (www.nitrc.org/projects/conn) (**Fig S7**).[70]

<u>Mediation Analysis</u>

We conducted a mediation analysis to test whether our connectivity results were mediated by the previously reported lesion overlap with the insula [13,15] (**Fig S8**). To maintain consistency with the original reports, we used categorical intersection with the insula (yes / no) as previously described and recorded for each lesion [13,15]. We next computed the similarity of each patient's lesion connectivity to our addiction remission map using spatial correlation, converted to a normal distribution using Fisher's r to z transform. This results in a single number for each lesion that reflects connectivity of the lesion location to our addiction remission network. Using the MATLAB Mediation Toolbox by Tor Wager (https://github.com/canlab/MediationToolbox),[71] we conducted a mediation analysis with lesion connectivity as the primary predictor, addiction remission as the primary outcome, and insula overlap as the mediator. We hypothesized that the effect of connectivity on addiction remission would not be mediated by insula overlap. For completeness, we also tested whether connectivity to our addiction remission network might mediate the previously reported association between insula damage and addiction remission.

<u>Behavioral effects in the Iowa smoker cohort</u>

After occurrence of the lesion, a subset of the patients in the Iowa cohort underwent neuropsychiatric testing, including Wechsler Adult Intelligence Scale III, Minnesota Multiphasic Personality Inventory and Trail Making Test Part B. To investigate if addiction remission was related to other neuropsychiatric changes, such as frontal lobe dysfunction or depression, we compared patients who remitted to patients who did not quit smoking using independent samples t-test (**Table S4**). To ensure that these subsets were representative of the whole sample, we re-computed our addiction remission map using only the subset of patients that underwent any (n = 57) or all (n = 34) neuropsychological assessments. Similarity with the addiction remission map computed using these subsamples and the original addiction remission map computed using the full sample (n = 129) was assessed using spatial correlation (**Fig S9**).

<u>Generalizability: Alcoholism risk scores</u>

Brain lesions (n = 186) were collected from the Vietnam Head Injury Study[41] for patients that had completed the MacAndrew Alcoholism Scale (MAC) (**Table S6**),[72] a subscale of the Minnesota Multiphasic Personality Inventory which measures personality characteristics that predispose to alcoholism.[73] Patients only completed this scale once, years after their focal brain injury.[41] All brain lesions had been previously traced based on CT scans and transformed to the MNI atlas, as described earlier.[69] Connectivity with each lesion location was computed as described above in "lesion network mapping." Connections correlated with MAC score were identified on a voxel-wise basis using Pearson's correlation. This process yielded a map of connections with the lesion location correlated with decreased risk of alcoholism. This correlation map was

converted to a normal distribution using Fisher's r to z transform. This alcoholism risk map was then compared with our addiction remission map using spatial correlation. To assess if the spatial similarity was higher than expected by chance, the spatial correlation coefficient was compared to a null distribution of spatial correlation coefficients calculated using permutation.[50] Specifically, our addiction remission map was re-generated with randomly permuted group labels (addiction remission versus non-quitters), our alcoholism risk map was regenerated with randomly permuted MAC scores, and the spatial correlation between the two maps was computed (1,000 iterations). The permutation method has been described in more detail earlier.[50] To determine if similar results could be obtained using lesion location alone (i.e. without using connectivity), we repeated this permutation analysis using VLSM maps rather than the lesion networks.

To ensure that the results were specific to reduced addiction risk, we repeated the analysis using the 10 main scales of the MMPI.[73] We again used spatial correlation and permutation to test whether the maps generated using each of these MMPI scales were more similar to our addiction remission network than would be expected by chance. As a direct test of specificity, we also compared the spatial correlation obtained using our alcoholism risk map to the spatial correlation obtained using these other 10 MMPI scales. The MMPI is designed such that these 10 scales are independent of one another, however we also orthogonalized the data to ensure that this was the case in our sample (results were similar with or without orthogonalization). Spatial correlations were converted to a normal distribution using Fisher's r to z transform. We then compared the spatial correlation with the addiction risk map to the distribution of spatial correlations with these other 10 maps using a one-sample two-tailed t-test.

To evaluate specificity beyond the domains measured by the MMPI, we analyzed 27 other behavioral variables available in this dataset including the Beck Depression Inventory (BDI), mini mental status exam (MMSE), and 25 subscales of the neurobehavioral rating scale (2 subscales were excluded because more than 90% of the values were zero). For each variable, we repeated the same analysis we performed for addiction risk, again assessing spatial similarity using permutation. We the qualitatively compared the spatial correlation coefficients for these 27 variables to the spatial correlation with alcoholism risk. Note that due to collinearity of these 27 measurements (e.g. depression scores from neurobehavioral rating score and BDI), we could not perform a direct statistical specificity analysis similar to the above analysis using the different MMPI scales (which are independent of one another).

Generalizability: Case reports of lesions resulting in addiction remission to other substances

A literature search for case reports of lesions resulting in remission of substance use disorders was conducted in PubMed at the end of 2017 using the following searches: "substance use disorder" AND "lesion" AND "brain" which resulted in 321 hits, and "substance use disorder" AND "injury" AND "brain" which resulted in 1577 hits. Resulting entries were then screened for cases where brain lesions resulted in addiction remission (abstinence from drug seeking and craving) in patients with non-nicotine substance use disorders. This search identified three cases where an acute brain lesion resulted in remission of addiction to substances other than nicotine (**Fig. S11**).[74-76] The lesion location and connectivity in each case was defined as described above. The spatial correlation between these connectivity maps and the maps derived from smoking patients were computed and statistically compared between lesions resulting in addiction remission and not quitting using Mann-Whitney U-test.

Electric field models of the Brainsway H4 and H7 TMS Coils

The Brainsway TMS device using H4 coil was cleared by the US Food and Drug Administration (FDA) for short-term smoking cessation in late 2020. This coil was designed to target multiple brain regions. TMS acts by creating a rapidly changing electric field (E-field) in the brain via electromagnetic induction.[11,12] The E-field distribution in the brain is determined by several factors, including the coil shape and head anatomy, and can be computationally modelled.[12] The 3D E-field model of the FDA approved coils in the MNI space was obtained from the manufacturer (Brainsway, NJ, USA). The field maps were overlaid on the MNI brain and surface projection of the volumetric map is created with Mango (version 4.1, https://rii.uthscsa.edu/mango/) (**Fig. S12**). The E-field intensity is displayed as a percentage relative to the peak intensity in the brain.

Supplementary figures

Figure S1. Smoker cohort lesions.
Lesions that resulted in addiction remission for smoking (A, n = 34). Lesions in patients who did not quit smoking (B, n = 69). Lesions in patients who quit but did not remit (C, n = 26). Each slice represents a different patient. There was no difference in lesion size between the groups (one-way ANOVA, F = 1.78, p = 0.17).

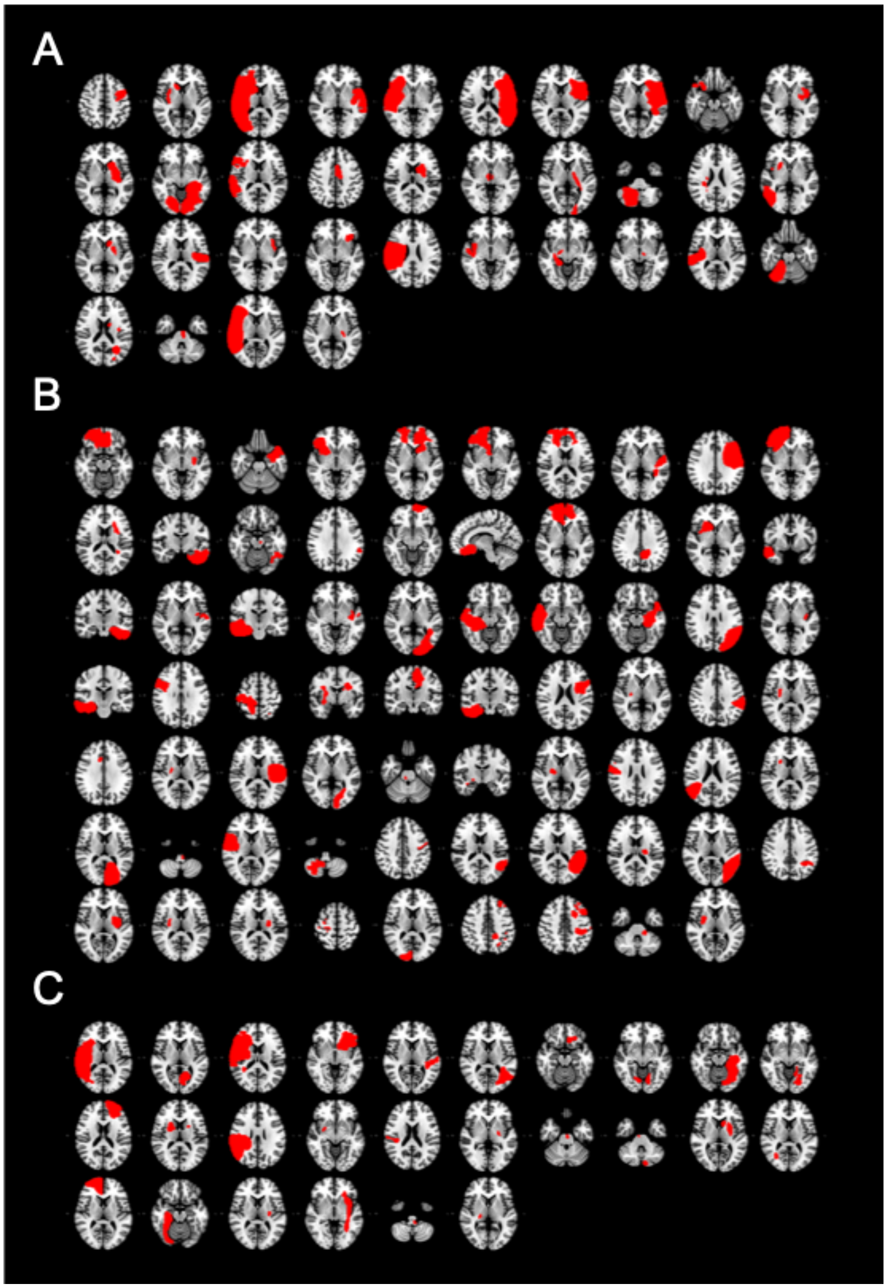

Fig. S2. Lesion locations and voxel-lesion symptom mapping (VLSM) results.
Lesion locations from actively daily smokers were combined across our two cohorts (n = 129) and overlaid to produce a lesion coverage map for all lesions (A) or lesions associated with a specific clinical outcome (B). Voxel intensity reflects the number of lesions intersecting each brain voxel. VLSM z-maps were constructed to identify voxels most associated with addiction remission for the Iowa dataset (C, left), Rochester dataset (C, middle) and both datasets together (C, right). Warm colors denote voxels more likely to be lesioned in the addiction remission group while cool colors denote voxels more likely to be lesioned in non-quitters. Addiction remission VLSM z-maps constructed using the Iowa (C, left) and Rochester (C, middle) cohorts were not more similar than what would be expected by chance. As there were no voxels significantly associated with addiction remission, the VLSM maps are shown unthresholded.

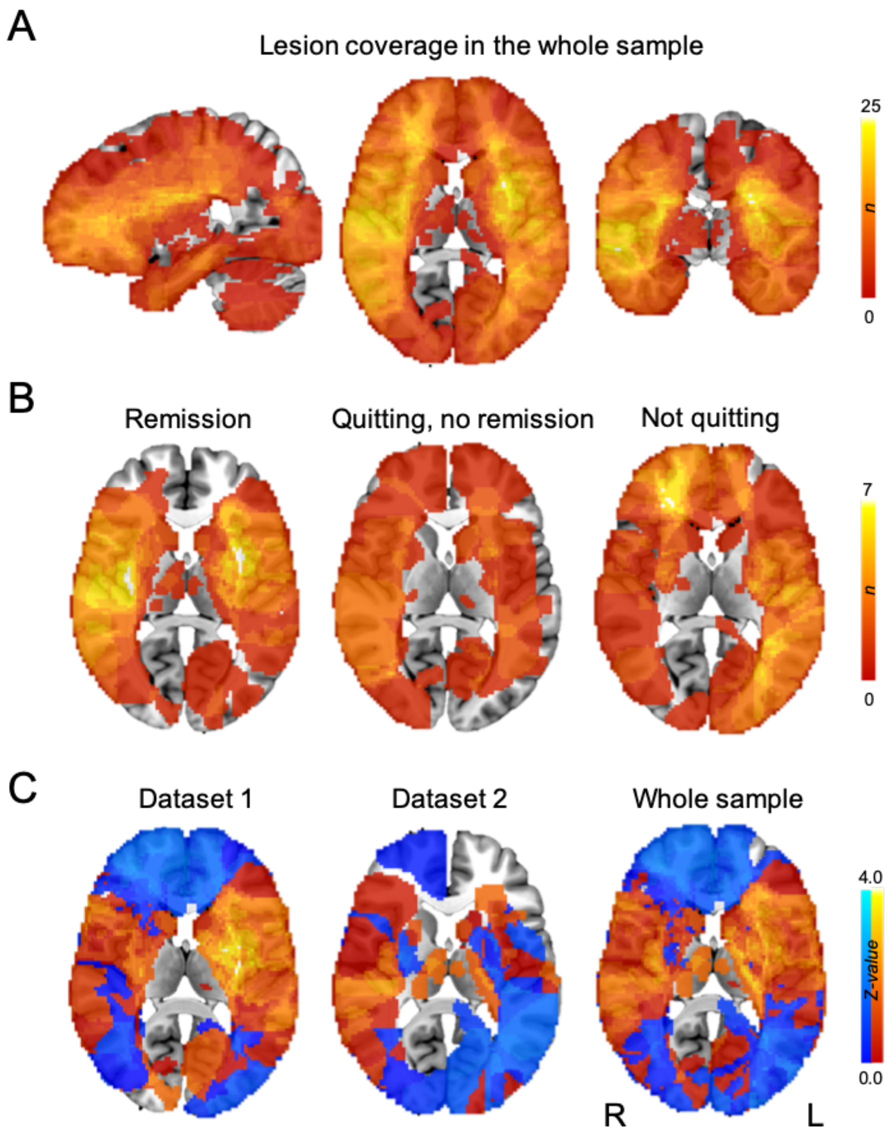

Fig. S3. Addiction remission network maps are reproducible when computed in each dataset independently. The addiction remission network map for the Iowa dataset (A) was similar to the addiction remission network map for the Rochester dataset (B). The spatial correlation between the two network maps (r = 0.58) was higher than expected by chance (permutation analysis, one-sided p = 0.03).

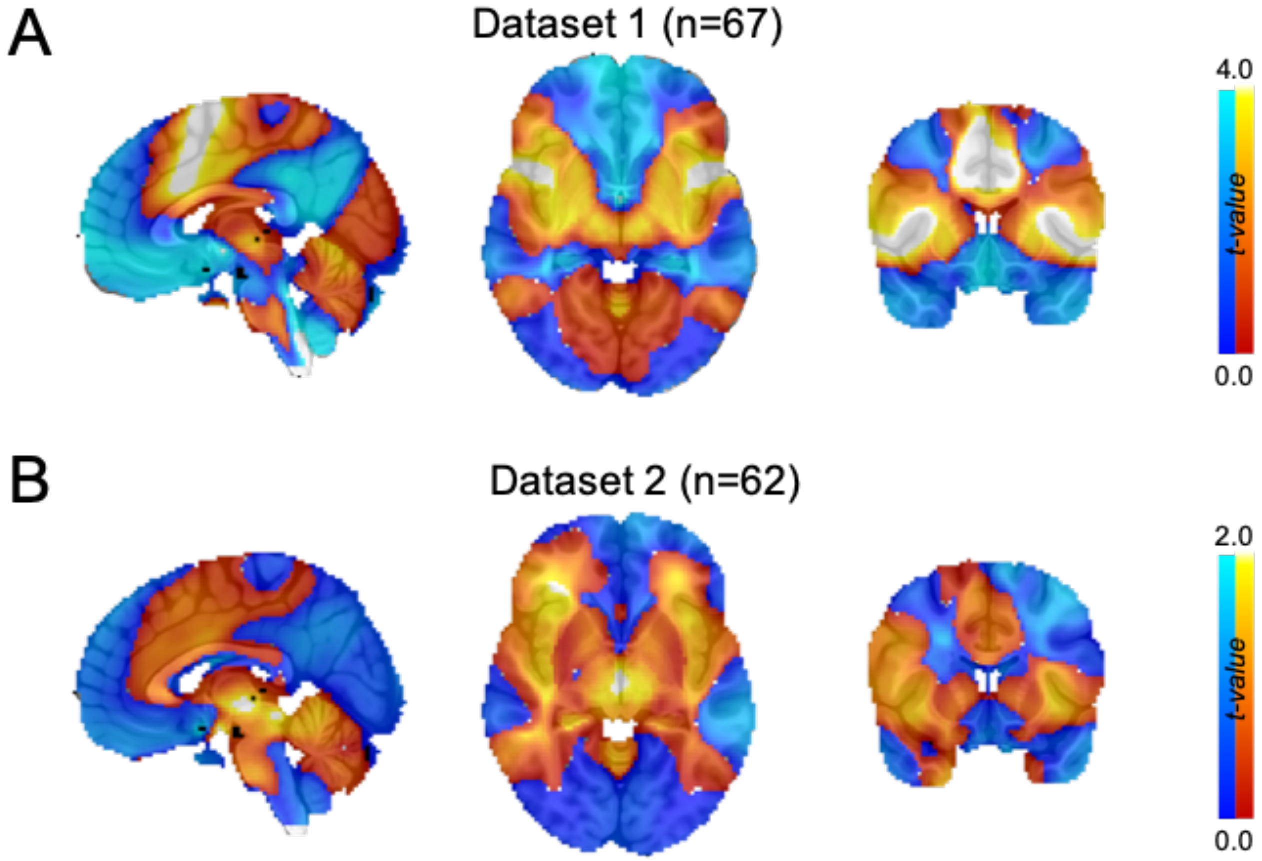

Fig. S4. Lesion network overlap results.
Individual lesion networks were thresholded, binarized, then overlaid for lesions associated with remission of addiction (A) and not quitting (B). Voxel value denotes the number of lesion locations functionally connected to that voxel (displayed at a threshold 75% of lesion locations). Lesion network overlap is much higher for lesions associated with remission of addiction (A) compared to lesions from patients who did not quit smoking (B), showing greater consistency in the former compared to the latter.

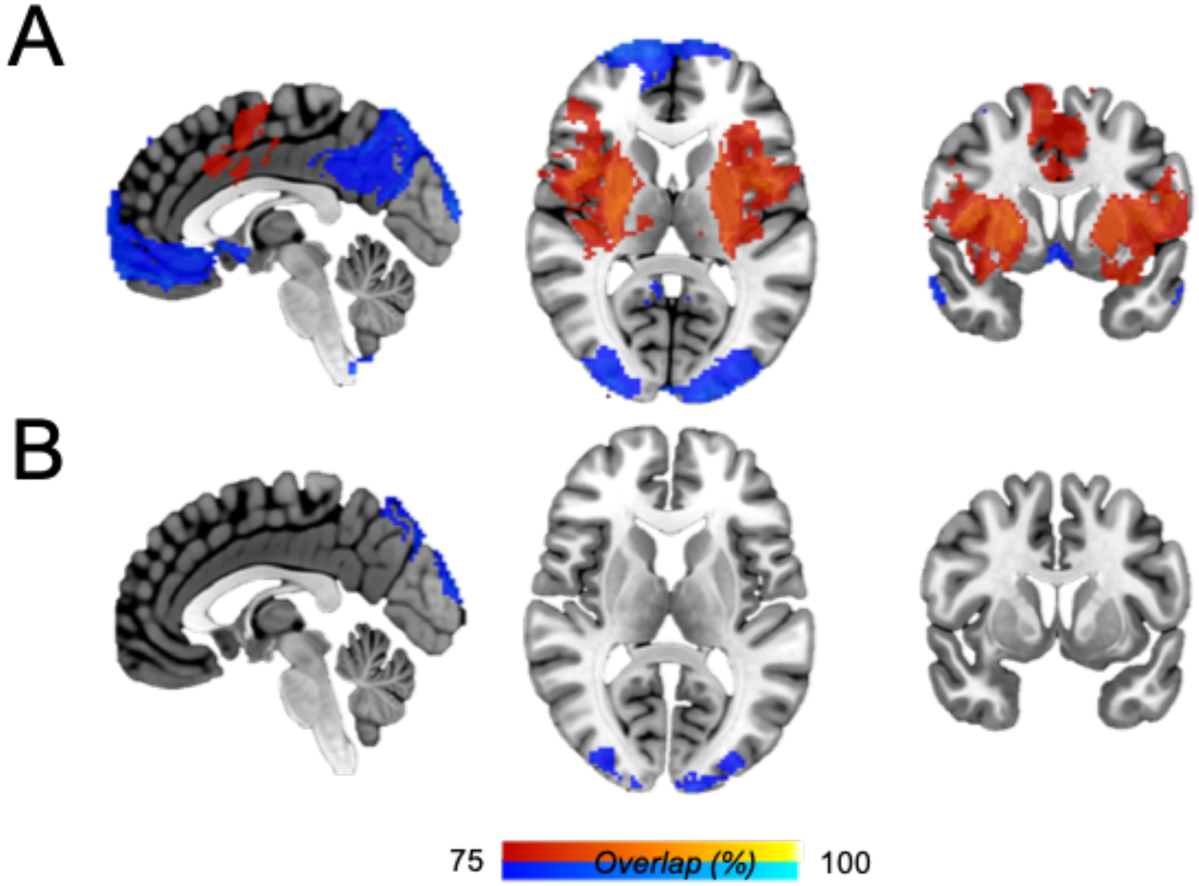

Fig. S5. Results are robust to different cut-offs for active daily smoking
A. Original addiction remission map computed with the full sample (n=129). B. Addiction remission map computed from patients smoking at least 5 cigarettes per day (n=121) was almost identical to the original map (spatial correlation r = 0.99). C. Addiction remission map computed from patients smoking at least 20 cigarettes per day (n=86) was again nearly identical to the original map (spatial correlation r = 0.98).

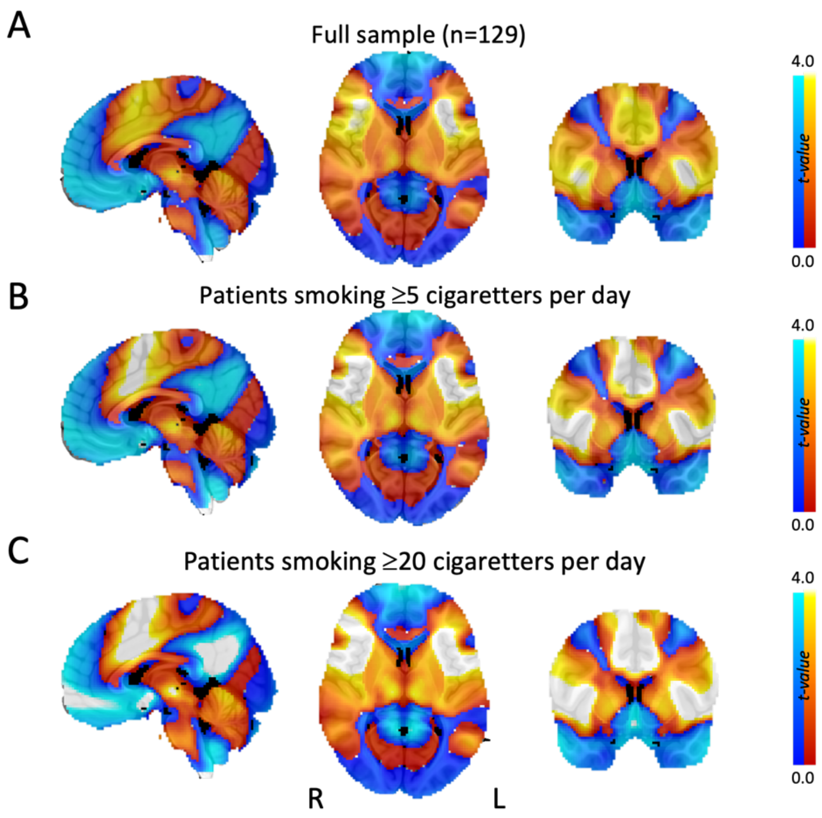

Fig. S6. Addiction remission network results are robust to methodological choices.
Our addiction remission network map from our primary analysis (A) remained unchanged when including lesion size (B), proportion of gray/white matter in the lesion (C) or patient age (D) as a covariate, excluding lesions other than strokes (E), or only including lesion patients with MRI (F). Maps are shown unthresholded to facilitate comparison. Spatial correlation coefficients between our original addiction remission network map (A) and the maps in B, C, D, E and F were r=0.99, r=0.99, r=0.999, r=0.98 and r=0.94, respectively.

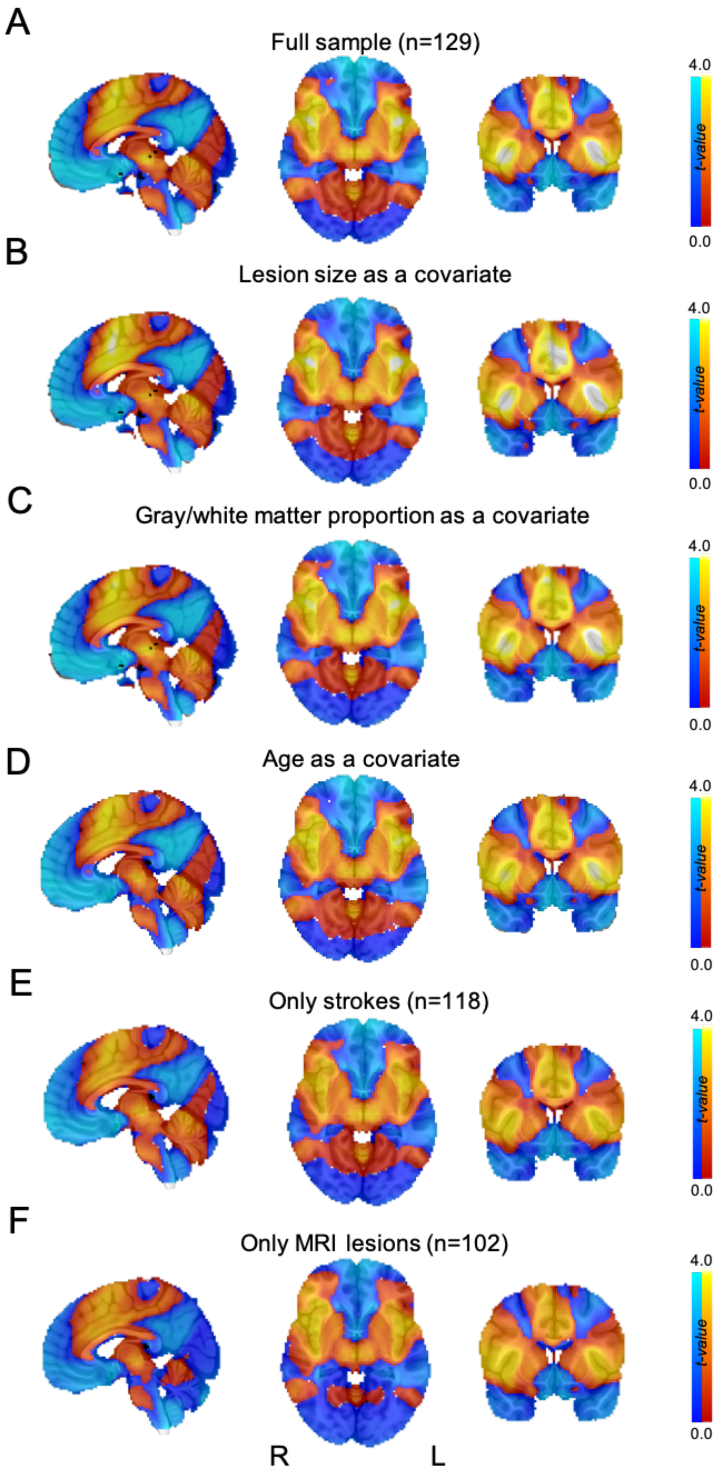

Fig. S7. Addiction remission network results are robust to the connectome.
Our addiction remission network map from our primary analysis using an rs-fcMRI dataset from 1,000 healthy volunteers (A) remained unchanged compared to using rs-fcMRI dataset from 126 smokers with spatial correlation coefficient r=0.94 (B), or to healthy volunteer connectome processed without global signal regression (r=0.94, C). The significant clusters surviving correction for multiple comparisons with threshold free cluster enhancement (P_{FWE}<0.05) were also almost identical (D-F). Test-retest reliability was significantly better for the normative connectome [mean (SD) r^2 0.98 (0.01)] compared to the smoker connectome [0.81 (0.10), two-sided paired t-test p<0.001] and normative connectome size-matched to the smoker connectome [0.90 (0.05), two-sided paired t-test p<0.001].

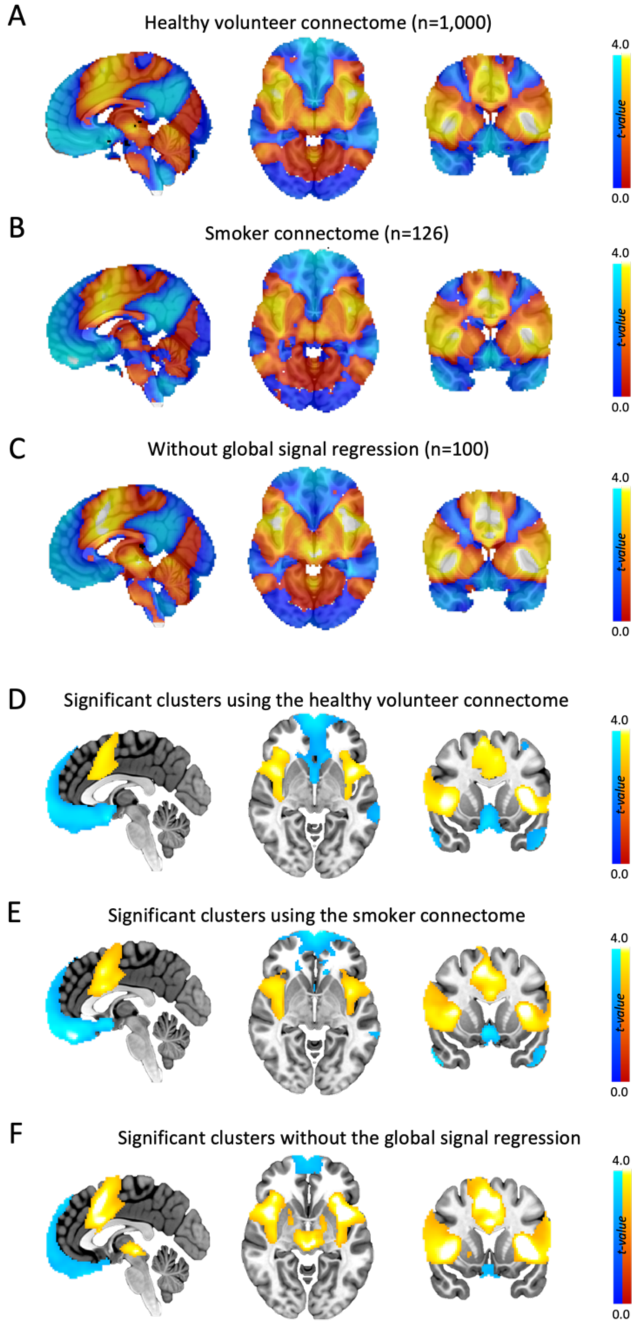

Fig. S8. Mediation analysis
Lesion overlap with the insula was significantly predictive of addiction remission, as was lesion connectivity. However, the effect of lesion connectivity on addiction remission was not mediated by the effect of lesion overlap with the insula (A). Interesting, the previously noted association between insula damage and addiction remission may be mediated by connectivity to our addiction circuit (B).

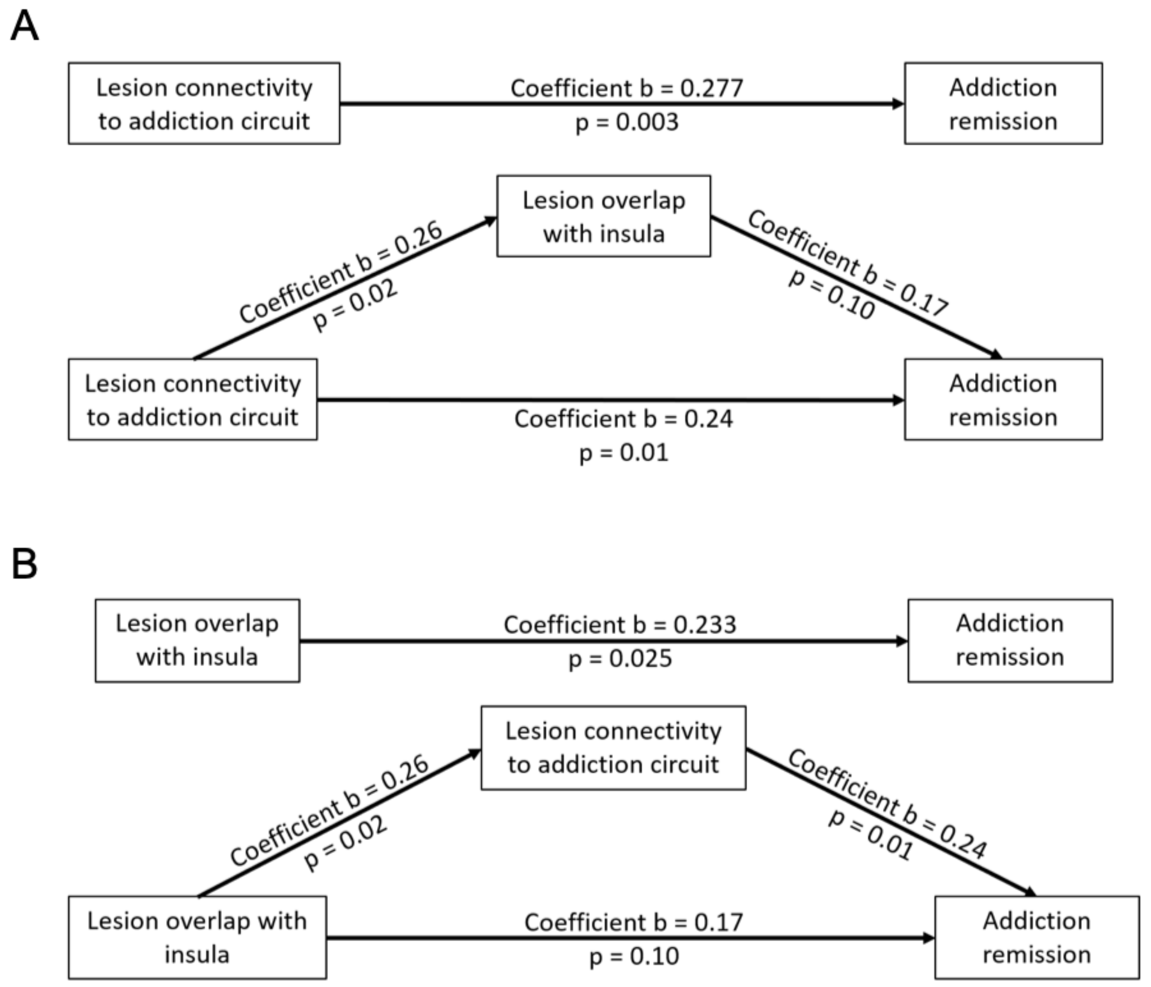

Fig. S9. Subsamples with neuropsychological assessment are representative of the full sample.
Our addiction remission network map from our primary analysis (A) remained unchanged when limiting the analysis to patients who underwent any (B) or all (C) neuropsychological assessments. Maps are shown unthresholded to facilitate comparison.

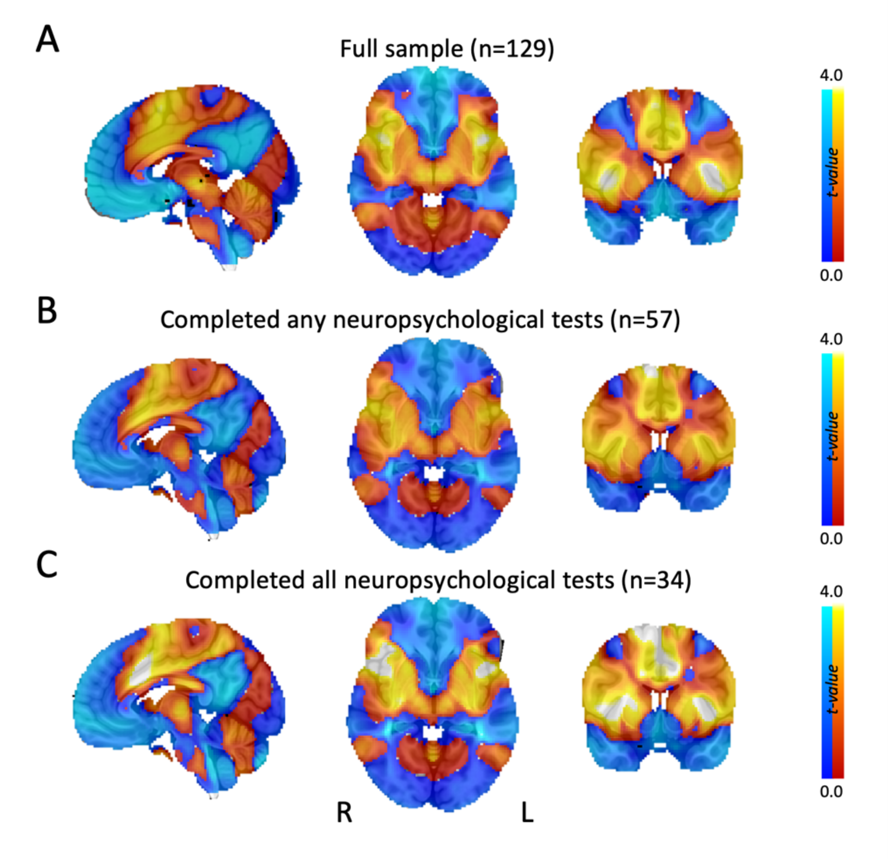

Figure S10. Structural connections related to addiction remission
A data-driven analysis identified two white matter tracts in the left fronto-insular cortex significantly associated with addiction remission (A, left panel, blue). Compared to the addiction remission network derived using the functional connectome (A, middle panel), there was clear overlap between the location of these white matter tracts and the left fronto-insular peak of our addiction remission network (A, right panel). In a complimentary analysis, we computed structural connectivity between each lesion location and our addiction remission network, finding high connectivity for lesions associated with addiction remission (B, single example shown) and weak connectivity for lesions from non-quitter (C, single example shown). Panels B and C display the lesion location (red, left panel), structural connectivity with the lesion location (green, middle panel), and intersection with our addiction remission network derived using the functional connectome (orange/yellow, right panel). This intersection was used to compute a "disconnectome score" for each lesion. At the group-level, lesions associated with addiction remission (n=34) showed significantly higher structural disconnectome scores compared to non-quitters (n=69) (Welsch's one-sided t-test with unequal variances, p = 0.003). (D). Data in panel D are presented as mean (bar) and SD (whiskers) with individual patient values (dots). **P<0.01

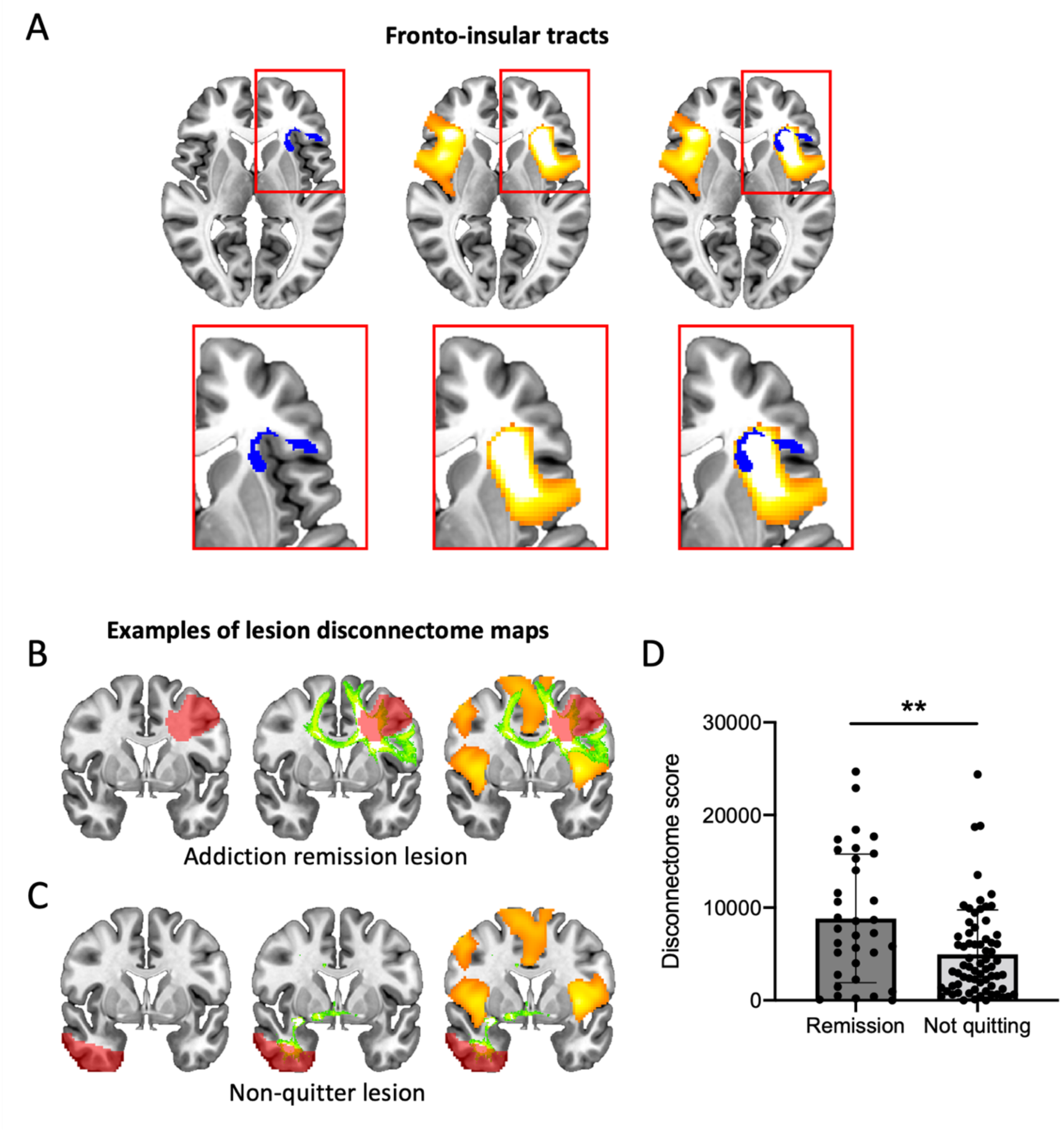

Fig. S11. Generalizability of findings to other substance use disorders.
We identified case reports of brain lesions that disrupted polysubstance addiction (Case #1), addiction to opioids and alcohol (Case #2), and addiction to alcohol and nicotine (Case #3) (A). For each case we show the lesion location (left, red) and connectivity with the lesion location (right). The green overlay shows the outline of significant regions from our addiction remission network (B, see **Figure 2D**). Each of these connectivity maps were more similar to the connectivity maps of lesions disrupting smoking addiction than to maps of lesions associated with not quitting smoking (Mann-Whitney U-test $p < 0.05$ in all cases).

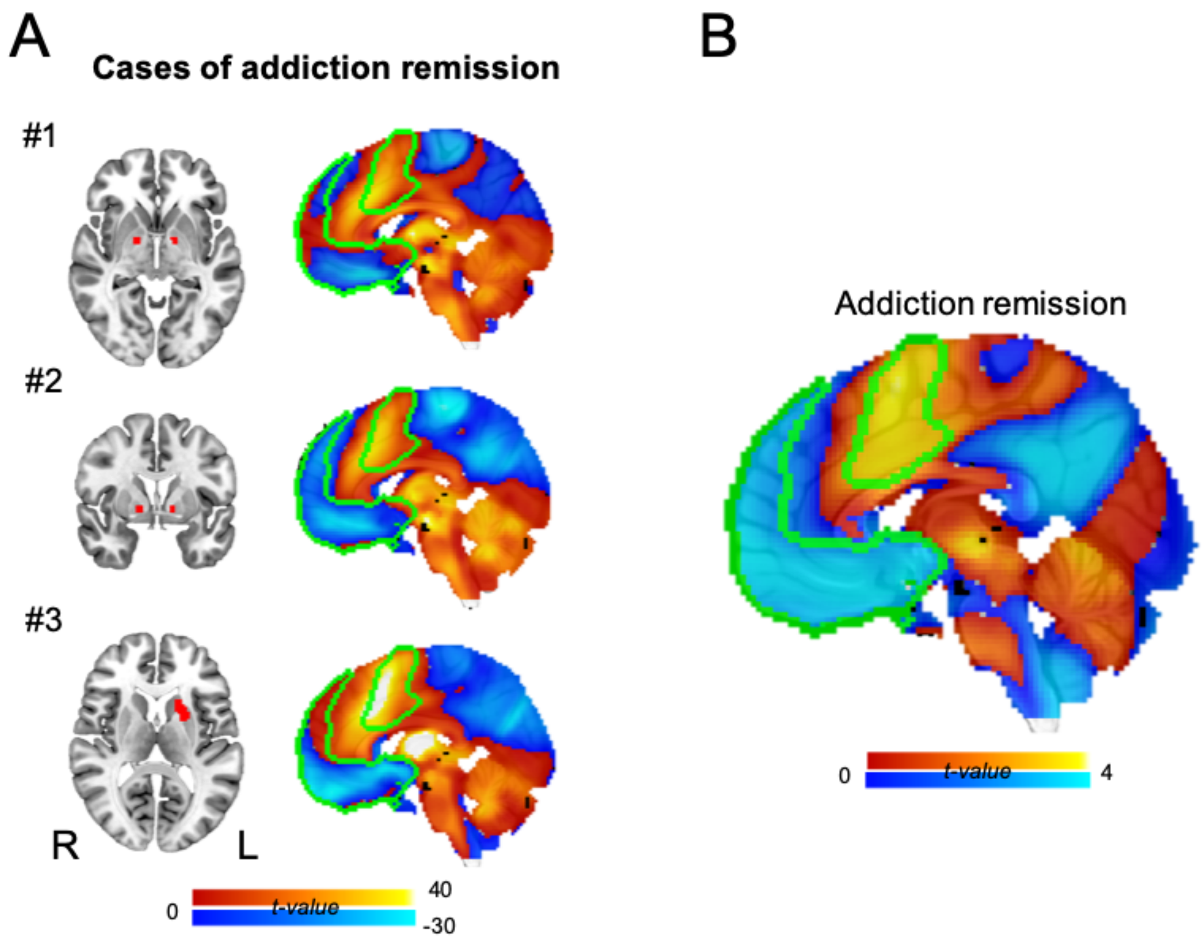

Fig. S12. Brainsway coil electric field (E-field) models.
E-field of the H4 coil for smoking cessation (A) and H7 coil for treatment of alcoholism. For clarity and to highlight the entire spectrum of the E-field intensities, a different color scale was chosen for this supplementary figure compared to Fig. 4 (bottom row). Note that the E-field models do not cover the back of the brain. However, the E-field intensities in the brain regions outside the E-field models are likely to be very low. A threshold of 20% of the maximum E-field is used for the illustration.

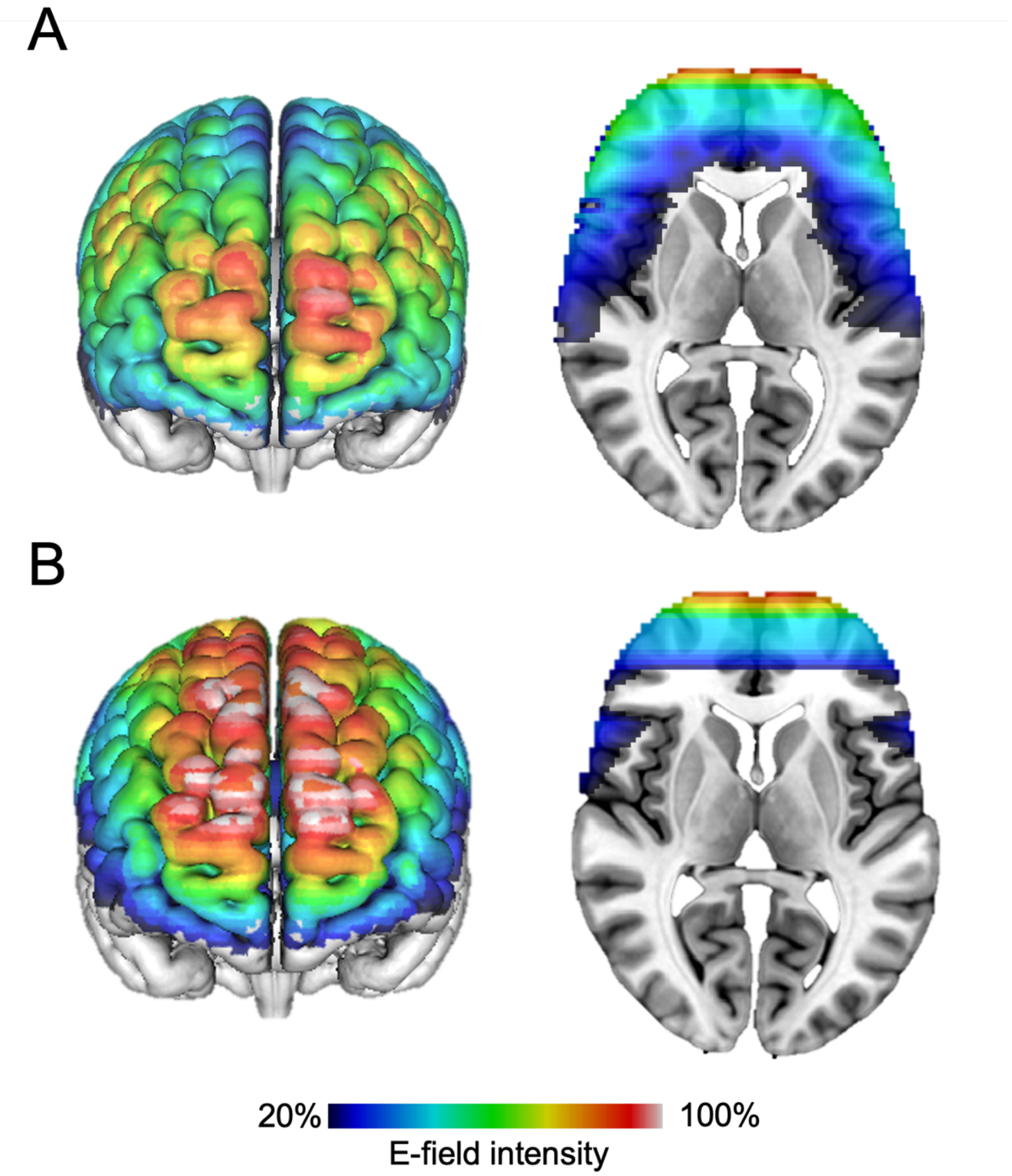

Supplementary tables

Table S1. Demographical data for smoker lesion datasets.

	Dataset 1 (Iowa)	Dataset 2 (Rochester)	Total
**n**	67	62	129
**sex (m/f)**	40/27	38/24	78/51
**age [mean (SD) y]**	53 (11)	59 (12)	56 (12)
**Strokes**	56 (84%)	62 (100%)	118 (91%)
**Modality (MRI/CT)**	50/17	52/10	102/27
**Lesion size (cm^3)**	63.6 (68.4)	22.9 (35.5)	44.0 (58.6)
**Proportion of gray/white matter**	0.31 (0.42)	0.03 (0.61)	0.17 (0.54)

Table S2. **Significant clusters in the addiction remission network map.**

Disruption of addiction > not quitting						
	Peak xyz coordinates			Max T	Voxels	Cluster location(s)
Cluster 1	38	30	6	4.07	4413	R Operculum/insula/lateral frontal cortex
Cluster 2	-34	26	8	4.37	3342	L Operculum/insula/dorsolateral frontal cortex
Cluster 3	6	12	60	3.93	2929	B Cingulate/paracingulate/dorsomedial frontal cortex
Cluster 4	68	-30	34	3.69	905	R Temporoparietal junction

Disruption of addiction < not quitting						
	Peak xyz coordinates			Max T	Voxels	Cluster location(s)
Cluster 1	2	6	-18	4.03	16613	B medial prefrontal cortex, subcallosal cingulate, medial and lateral temporal lobe
Cluster 2	64	-8	-28	3.54	1947	R Lateral temporal lobe

The clusters are shown in Fig. 2D and coordinates are reported in MNI space (mm) and cluster sizes in voxels (2 x 2 x 2mm). Many clusters included multiple neuroanatomical regions which are listed under cluster location(s). R = right, L = left, B = bilateral.

Table S3. Demographical data for the smoker connectome subjects

**n**	126
**Sex (m/f)**	68/58
**Age [mean (SD) y]**	33.7 (10.0)
**Cigarettes Per Day [mean (SD) y]**	16.7 (6.5)
**Years Smoked [mean (SD) y]**	16.1 (9.6)

Table S4. Neuropsychological test scores

	Remission	Quitters (no remission)	Non-quitters	N of subjects	p-value[1]
Wechsler Adult Intelligence Scale III (WAIS-III)					
Verbal IQ (complete)	103.1 (11.2)	98.2 (13.2)	100.6 (19.4)	n1=8,n2=13,n3=25	0.65
Vocabulary	11.1 (2.6)	9.9 (3.1)	10.2 (3.4)	n1=9,n2=13,n3=25	0.44
Similarities	10.5 (3.0)	10.6 (2.7)	9.7 (3.1)	n1=11,n2=13,n3=33	0.46
Arithmetic	8.4 (2.7)	10.2 (2.5)	9.7 (3.3)	n1=11,n2=13,n3=35	0.21
Digit span	7.6 (2.9)	8.3 (1.8)	9.4 (3.0)	n1=11,n2=13,n3=37	0.10
Information	10.6 (3.1)	9.6 (2.8)	9.7 (3.1)	n1=11,n2=13,n3=33	0.40
Comprehension	12.3 (2.1)	9.9 (3.8)	10.9 (3.7)	n1=9,n2=13,n3=28	0.17
Letter-number sequencing	8.6 (2.9)	8.7 (2.2)	8.9 (4.0)	n1=9,n2=12,n3=27	0.77
Performance IQ (complete)	103.8 (15.6)	94.8 (12.3)	99.9 (14.4)	n1=9,n2=13,n3=25	0.53
Picture completion	10.8 (4.0)	8.8 (3.3)	11.0 (3.3)	n1=9,n2=13,n3=25	0.53
Digit symbol-coding	8.7 (3.6)	7.6 (2.2)	8.4 (2.7)	n1=9,n2=13,n3=35	0.88
Matrix reasoning	9.9 (2.7)	9.8 (3.0)	10.9 (2.3)	n1=14,n2=13,n3=33	0.24
Block design	9.0 (2.6)	9.9 (2.8)	10.6 (2.3)	n1=13,n2=13,n3=35	0.06
Picture arrangement	9.5 (3.5)	10.2 (2.6)	9.4 (2.6)	n1=13,n2=13,n3=28	0.87
Object assembly	9.1 (2.5)	8.4 (1.5)	10.5 (2.7)	n1=9,n2=9,n3=17	0.21
Symbol search	10.7 (2.5)	8.4 (3.1)	9.2 (3.0)	n1=9,n2=12,n3=26	0.16
Full scale IQ (complete)	104.9 (12.5)	96.3 (12.4)	99.7 (24)	n1=8,n2=13,n3=24	0.37
Processing speed index	97.9 (16.5)	88.4 (12.6)	91.8 (16.2)	n1=9,n2=12,n3=25	0.36
Perceptual-organization index	101.4 (16.5)	96.4 (12.6)	104.7 (13.7)	n1=12,n2=13,n3=26	0.55
Verbal comprehension index	109.0 (12.0)	100.2 (14.4)	100.4 (17.7)	n1=8,n2=13,n3=25	0.14
Working memory index	88.4 (15.1)	94.1 (8.8)	96.3 (19.1)	n1=9,n2=12,n3=27	0.23
Minnesota Multiphasic Personality Inventory (MMPI)					
Hypochondriasis	63.8 (10.1)	58.7 (15.5)	62.7 (15.5)	n1=8,n2=11,n3=20	0.86
Depression	64.1 (14.0)	58.5 (16.1)	61.3 (11.5)	n1=8,n2=11,n3=20	0.58
Hypomania	51.3 (7.9)	58.9 (11.4)	52.3 (11.7)	n1=8,n2=11,n3=20	0.83
Social introversion	55.4 (11.0)	55.7 (12.7)	55.6 (8.5)	n1=8,n2=11,n3=20	0.95
Hysteria	61.0 (10.1)	58.9 (11.4)	57.8 (14.5)	n1=8,n2=11,n3=20	0.58
Psychopathic deviance	55.3 (10.9)	55.0 (13.4)	54.9 (14.1)	n1=8,n2=11,n3=20	0.94
Masculinity / feminity	53.8 (12.6)	51.9 (8.4)	50.1 (12.0)	n1=8,n2=11,n3=20	0.48
Paranoia	58.3 (17.0)	51.8 (12.6)	57.5 (14.2)	n1=8,,n2=11,n3=20	0.91
Psychastenia	61.4 (15.2)	58.5 (19.6)	56.8 (14.0)	n1=8,n2=11,n3=20	0.45
Schizophrenia	63.3 (12.2)	61.2 (19.9)	59.8 (17.3)	n1=8,n2=11,n3=20	0.61
Beck Depression Inventory (total)	11.2 (6.7)	9.3 (9.2)	11.8 (9.6)	n1=9,n2=10,n3=31	0.85
Trail Making Test Part B (sec)	91.4 (77.8)	88.5 (44.6)	94.3 (41.9)	n1=9,n2=13,n3=33	0.88

Mean (SD) values for behavioral tests in the Iowa cohort. Higher scores in WAIS-III and lower in Tmt B indicate better performance, and higher scores in MMPI stronger trait/symptoms. n1 = subjects with smoking addiction remission. n2= subjects who quit smoking but did not fulfill the criteria for addiction remission. n3 = subjects who did not quit smoking. [1]Two-sided independent samples t-test remission vs non-quitters.

Table S5. White matter tracts associated with addiction remission

	Remission	Not quitting	P(uncorrected)	P(FWE corrected)
**Fronto-insular tract 1 left**	0.035 (0.064)	0.005 (0.029)	0.001	**0.04**
**Fronto-insular tract 2 left**	0.077 (0.141)	0.014 (0.066)	0.001	**0.04**
Fronto-insular tract 5 left	0.312 (0.444)	0.146 (0.317)	0.004	n.s.
Corticospinal tract left	0.496 (0.483)	0.292 (0.422)	0.004	n.s.
Fronto-insular tract 4 left	0.320 (0.451)	0.171 (0.346)	0.009	n.s.
Pons left	0.546 (0.483)	0.356 (0.457)	0.01	n.s.
Fronto-insular tract 3 left	0.207 (0.306)	0.105 (0.227)	0.01	n.s.
Frontal commissural tract	0.634 (0.425)	0.412 (0.463)	0.01	n.s.
Frontal inferior longitudinal tract left	0.229 (0.366)	0.106 (0.254)	0.02	n.s.
Frontal Aslant tract left	0.330 (0.451)	0.186 (0.361)	0.02	n.s.
Anterior thalamic projections left	0.484 (0.471)	0.317 (0.431)	0.02	n.s.
Hand inferior U tract left	0.235 (0.392)	0.113 (0.297)	0.02	n.s.
Frontal superior longitudinal tract left	0.235 (0.394)	0.114 (0.290)	0.04	n.s.
Face U tract left	0.140 (0.266)	0.065 (0.184)	0.05	n.s.

White matter tracts showing higher disconnection probability in patients with addiction remission compared to patients who did not quit smoking (Aspin-Welch's one-sided test, uncorrected $p < 0.05$). Family-wise error (FWE) correction for multiple comparisons was calculated using permutation analysis of linear models (PALM) analogous to the voxelwise analyses. Mean (SD) values of tract disconnection probability are shown. n.s. = not significant ($p > 0.05$).

Table S6. Demographical data for the Vietnam Head Injury Dataset

**n**	168
**Sex (m/f)**	168/0
**Age at lesion [mean (SD) y]**	20.3 (2.8)
**Age at testing [mean (SD) y]**	58.3 (3.1)
**Smokers**	70 (42%)

Table S7. Regions whose connectivity profile best matches the addiction remission network map.

	Positive connectivity					
	Peak xyz coordinates			Max R	Voxels	Main location(s)
Cluster 1	-32	24	10	0.874	1664	L Operculum/insula
Cluster 2	46	8	8	0.871	2251	R Operculum/insula
Cluster 3	2	10	54	0.863	1977	B Paracingulate/cingulate/dorsomedial frontal cortex
Cluster 4	-52	-36	30	0.818	697	L Temporoparietal junction
Cluster 5	52	-30	36	0.813	753	R Temporoparietal junction

	Negative connectivity					
	Peak xyz coordinates			Min R	Voxels	Main location(s)
Cluster 1	2	70	4	-0.829	1223	B Medial fronto-polar cortex/ventromedial prefrontal cortex
Cluster 2	64	-8	-22	-0.804	519	R temporal cortex
Cluster 3	-66	-18	-18	-0.793	556	L temporal cortex
Cluster 4	-50	-70	34	-0.779	382	L parietal cortex
Cluster 5	0	-46	32	0.767	156	Posterior cingulate
Cluster 6	54	-62	32	0.766	153	R parietal cortex

The clusters are defined at r > 0.75. Coordinates are reported in MNI space (mm) and cluster sizes in voxels (2 x 2 x 2mm) for clusters with >100 contiguous voxels. The peaks with highest positive and negative r values are shown in **Fig. 4**. R = right, L = left, B = bilateral.
