# Supplementary material for: Brain lesions disrupting addiction map to a common human brain circuit
Source: Nat Med. 2022 Jun 13;28(6):1249–55. doi: 10.1038/s41591-022-01834-y (PMC9205767; doi:10.1038/s41591-022-01834-y)
Supplement: Supplementary file 2 — Reporting Summary [file 41591_2022_1834_MOESM2_ESM.pdf]

nature research

Corresponding author(s): Juho Joutsa, Michael D. Fox

Last updated by author(s): 04/15/2022

Reporting Summary

Nature Research wishes to improve the reproducibility of the work that we publish. This form provides structure for consistency and transparency in reporting. For further information on Nature Research policies, see our Editorial Policies and the Editorial Policy Checklist.

Statistics

For all statistical analyses, confirm that the following items are present in the figure legend, table legend, main text, or Methods section.

n/a	Confirmed	
☐	☒	The exact sample size (*n*) for each experimental group/condition, given as a discrete number and unit of measurement
☐	☒	A statement on whether measurements were taken from distinct samples or whether the same sample was measured repeatedly
☐	☒	The statistical test(s) used AND whether they are one- or two-sided<br>*Only common tests should be described solely by name; describe more complex techniques in the Methods section.*
☐	☒	A description of all covariates tested
☐	☒	A description of any assumptions or corrections, such as tests of normality and adjustment for multiple comparisons
☐	☒	A full description of the statistical parameters including central tendency (e.g. means) or other basic estimates (e.g. regression coefficient) AND variation (e.g. standard deviation) or associated estimates of uncertainty (e.g. confidence intervals)
☐	☒	For null hypothesis testing, the test statistic (e.g. *F*, *t*, *r*) with confidence intervals, effect sizes, degrees of freedom and *P* value noted<br>*Give P values as exact values whenever suitable.*
☒	☐	For Bayesian analysis, information on the choice of priors and Markov chain Monte Carlo settings
☒	☐	For hierarchical and complex designs, identification of the appropriate level for tests and full reporting of outcomes
☐	☒	Estimates of effect sizes (e.g. Cohen's *d*, Pearson's *r*), indicating how they were calculated

Our web collection on statistics for biologists contains articles on many of the points above.

Software and code

Policy information about availability of computer code

Data collection	Lesion network maps were constructed using in-house scripts in combination with public human connectome data, as described below.
Data analysis	All analyses were conducted using freely available codes and software, as described in the manuscript. The software include FSL (version 6.0), Niistat (version 1.0.20191216). The code for lesion connectivity analysis is freely available in Lead DBS software (www.lead-dbs.org). We have made a fully pre-processed version of our 1000 subject functional connectome publicly available [https://doi.org/10.7910/DVN/ILXIKS] created using the Brain Genomics Superstuct Project (GSP) data [https://doi.org/10.7910/DVN/25833], along with all of the code and parameters used to process the data [https://doi.org/10.5281/zenodo.4905738].

For manuscripts utilizing custom algorithms or software that are central to the research but not yet described in published literature, software must be made available to editors and reviewers. We strongly encourage code deposition in a community repository (e.g. GitHub). See the Nature Research guidelines for submitting code & software for further information.

Data

Policy information about availability of data

All manuscripts must include a data availability statement. This statement should provide the following information, where applicable:

- Accession codes, unique identifiers, or web links for publicly available datasets
- A list of figures that have associated raw data
- A description of any restrictions on data availability

De-identified lesion masks in MNI atlas space from our two primary datasets (Iowa and Rochester cohorts) are available from Harvard Dataverse (https://doi.org/10.7910/DVN/8BHHRS). Clinical and behavioral data from the lesion patients is available upon request, subject to the policies and procedures of the institution where each dataset was collected. Data requests should be sent to the corresponding authors.

Field-specific reporting

Please select the one below that is the best fit for your research. If you are not sure, read the appropriate sections before making your selection.

☒ Life sciences ☐ Behavioural & social sciences ☐ Ecological, evolutionary & environmental sciences

For a reference copy of the document with all sections, see nature.com/documents/nr-reporting-summary-flat.pdf

Life sciences study design

All studies must disclose on these points even when the disclosure is negative.

Sample size	Because there is no standard method for estimating sample size for this type of study, we attempted to identify as much data as was possible.
Data exclusions	All subjects that were available and matched the inclusion criteria (a brain lesion in the MRI/CT and outcome measures of interest) were included into this study, as described in the methods.
Replication	As outlined in the manuscript, we used rigorous statistical techniques to assess overall reproducibility across three different datasets incuded in the study. No replication attempts were unsuccessful.
Randomization	Rather than prospective randomization, this study capitalized on incidental variability of brain lesions. This incidental variability was presumed to be random.
Blinding	Blinding was not relevant because this was a secondary analysis of existing datasets and the investigators were not involved in the clinical outcome evaluation.

Reporting for specific materials, systems and methods

We require information from authors about some types of materials, experimental systems and methods used in many studies. Here, indicate whether each material, system or method listed is relevant to your study. If you are not sure if a list item applies to your research, read the appropriate section before selecting a response.

Materials & experimental systems

n/a	Involved in the study	
☒	☐	Antibodies
☒	☐	Eukaryotic cell lines
☒	☐	Palaeontology and archaeology
☒	☐	Animals and other organisms
☐	☒	Human research participants
☒	☐	Clinical data
☒	☐	Dual use research of concern

Methods

n/a	Involved in the study	
☒	☐	ChIP-seq
☒	☐	Flow cytometry
☐	☒	MRI-based neuroimaging

Human research participants

Policy information about studies involving human research participants

Population characteristics	Participants included adults who had incidental brain lesions. These data include 129 smokers with brain lesions (mean age 56 years, 51 females, 78 males), 168 participants from the Vietnam head injury dataset (mean age at testing 58 years, all males). Demographical information for each dataset are provided as supplementary material.
Recruitment	We included all subjects from relevant datasets that we were able to access. Each dataset had different recruitment parameters depending on the study type.
Ethics oversight	The study was approved by the local institutional review boards (Beth Israel Deaconess Medical Center #2018P000128 and Brigham and Women's hospital #2020P002987) and all subjects have provided written informed consent as part of the original study they were enrolled in.

Note that full information on the approval of the study protocol must also be provided in the manuscript.

Magnetic resonance imaging

Experimental design

Design type	Structural lesion locations defined on brain MRI and/or head CT combined with normative resting-state fMRI

Design specifications

Structural MRI or CT scans were used to localize lesions and/or stimulation sites. Resting-state fMRI data from a large connectome database (healthy volunteers n=1000, smokers n=126) were used to estimate connectivity of each site.

Behavioral performance measures

The main behavioral measure: smoking behavior after occurrence of the brain lesion (remission, quit smoking, did not quit smoking). Remission was defined as in Naqvi et al. (Science 2007). Other behavioral are described in the manuscript.

Acquisition

Imaging type(s)

Resting-state fMRI (n=1000 healthy controls, n=126 active daily smokers) and individualized structural lesion locations based on structural MRI or CT (n=315)

Field strength

3T

Sequence & imaging parameters

Normative (n=1000) resting-state fMRI acquisitionn parameters: repetition time (TR) = 3,000 ms, echo time (TE) = 30 ms, flip angle (FA) = 85°, 3 × 3 × 3-mm voxels, field of view (FOV) = 216, and 47 axial slices collected with interleaved acquisition and no gap between slices. Each functional run lasted 6.2 min (124 time points). One or two runs were acquired per subject (average of 1.7 runs).
Confirmatory dataset of active daily smokers (n=126): please see details in supplementary materials page 4.

Area of acquisition

Whole brain

Diffusion MRI ☐ Used ☒ Not used

Preprocessing

Preprocessing software

FreeSurfer + in-house preprocessing scripts, as in the GSP1000 dataset (details in Yeo et al, J Neurophysiol 2011)

Normalization

Nonlinear volume-based registration

Normalization template

MNI ICBM152

Noise and artifact removal

Low-pass temporal filtering, head-motion regression, global signal regression, and ventricular and white matter signal regression

Volume censoring

Motion regression

Statistical modeling & inference

Model type and settings

Lesion network mapping with voxel-wise partial least squares regression model (details described in manuscript).

Effect(s) tested

The main effect tested was the difference in lesion location connectivity between smokers who remitted vs. who did not quit smoking, controlling for dataset. Secondary analyses included correlations between lesion location connectivity and continous behavioral data, as described in the manuscript in detail.

Specify type of analysis: ☒ Whole brain ☐ ROI-based ☐ Both

Statistic type for inference
(See Eklund et al. 2016)

Treshold-free cluster enhancement (TFCE)

Correction

FWE

Models & analysis

n/a	Involved in the study
☐	☒ Functional and/or effective connectivity
☒	☐ Graph analysis
☒	☐ Multivariate modeling or predictive analysis

Functional and/or effective connectivity

Mean Pearson correlation across the normative dataset (n=1000) for each lesion location.
